# Polyamide nanofiltration membrane with highly uniform sub-nanometre pores for sub-1 Å precision separation

**DOI:** 10.1038/s41467-020-15771-2

**Published:** 2020-04-24

**Authors:** Yuanzhe Liang, Yuzhang Zhu, Cheng Liu, Kueir-Rarn Lee, Wei-Song Hung, Zhenyi Wang, Youyong Li, Menachem Elimelech, Jian Jin, Shihong Lin

**Affiliations:** 1 i-Lab and CAS Center for Excellence in Nanoscience, Suzhou Institute of Nano-Tech and Nano-Bionics, Chinese Academy of Sciences, 215123 Suzhou, P.R. China; 2 Department of Civil and Environmental Engineering, Vanderbilt University, Nashville, TN 37235-1831 USA; 3 Interdisciplinary Material Science Program, Vanderbilt University, Nashville, TN 37235 USA; 4 Institute of Functional Nano and Soft Materials, Soochow University, 215123 Suzhou, P. R. China; 5 R&D Center for Membrane Technology, Department of Chemical Engineering, Chung Yuan University, 32023 Chung Li, Taiwan; 6 Graduate Institute of Applied Science and Technology, National Taiwan University of Science and Technology, 10607 Taipei, Taiwan; 7 Department of Chemical and Environmental Engineering, Yale University, New Haven, CT 06520-8286 USA; 8 College of Chemistry, Chemical Engineering and Materials Science, Soochow University, 215123 Suzhou, P. R. China

**Keywords:** Chemical engineering, Polymers, Synthesis and processing

## Abstract

Separating molecules or ions with sub-Angstrom scale precision is important but technically challenging. Achieving such a precise separation using membranes requires Angstrom scale pores with a high level of pore size uniformity. Herein, we demonstrate that precise solute-solute separation can be achieved using polyamide membranes formed via surfactant-assembly regulated interfacial polymerization (SARIP). The dynamic, self-assembled network of surfactants facilitates faster and more homogeneous diffusion of amine monomers across the water/hexane interface during interfacial polymerization, thereby forming a polyamide active layer with more uniform sub-nanometre pores compared to those formed via conventional interfacial polymerization. The polyamide membrane formed by SARIP exhibits highly size-dependent sieving of solutes, yielding a step-wise transition from low rejection to near-perfect rejection over a solute size range smaller than half Angstrom. SARIP represents an approach for the scalable fabrication of ultra-selective membranes with uniform nanopores for precise separation of ions and small solutes.

## Abstract (web summary)

Separating molecules or ions with sub-Angstrom scale precision is important but technically challenging. Here, the authors demonstrate that precise solute-solute separation can be achieved using polyamide membranes formed via surfactant-assembly regulated interfacial polymerization.

## Introduction

Membranes capable of precise separation of ions and small molecules will have a transformative impact on the energy, water, chemical, and pharmaceutical industries^1–5^. Such separations require membranes with highly uniform pore sizes to obtain precise molecular sieving and solute differentiation^6,7^, which has been technically challenging to achieve. While the fabrication of highly precise membranes has been attempted recently using approaches such as stacking 2D nanomaterials or integrating aligned synthetic or biological channels^8–10^, no study thus far reported sub-Angstrom precision for separating sub-nanometer sized solutes in membrane filtration under applied pressure and crossflow. Moreover, these approaches face substantial technical challenges for scalable fabrication of defect-free membranes^11^.

Nanofiltration (NF) based on thin-film-composite polyamide (TFC-PA) membranes is a mature and energy-efficient technology for separating small solutes from liquid solvents^12–14^. The selective layer of polyamide-based NF membranes is formed by interfacial polymerization (IP) on a porous support. In a typical IP process, amine monomers in an aqueous solution diffuse into an organic solvent phase where they vigorously react with acyl chlorides at the water/organic interface via a Schotten-Baumann reaction^15,16^. Such uncontrolled diffusion and fast polymerization form a polyamide (PA) layer with multiscale heterogeneity and non-uniform pore sizes^17,18^. The mechanism of IP continues to attract heightened research interest due to the substantial use of TFC-PA membranes for desalination and water purification along with the lack of a thorough understanding of IP. While recent studies have explored different ways to improve the perm-selectivity of PA membranes^19–21^, achieving precise separation of ions and small molecules using PA membranes requires enhancing the pore size homogeneity, which entails a paradigm shift in engineering the PA active layer.

**Fig. 1:**
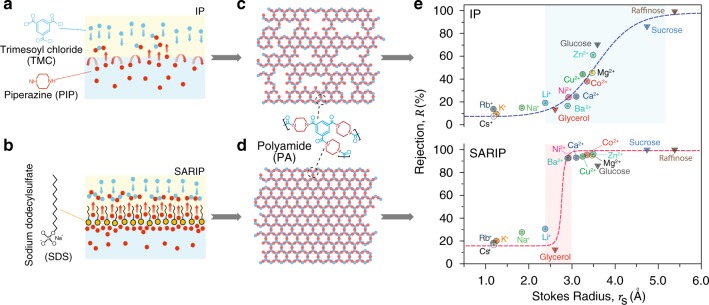
Conventional IP vs. SARIP. Schematic illustration of (**a**) the conventional IP and (**b**) SARIP. In both cases, PIP molecules in aqueous phase diffuse across the water/hexane interface to react with TMC in the hexane phase. In SARIP, SDS molecules added into the aqueous phase form a self-assembled dynamic network at the interface and regulate the interfacial transport of PIP. (**c**, **d**) Schematic illustrations of the PA active layer formed via conventional IP (top), which has a heterogeneous pore size distribution, and SARIP (bottom), which has a uniform pore size distribution. (**e**) Rejection of different solutes (circles for cations and inverted triangles for neutral organics) as a function of the Stokes radius for the PA membranes fabricated using conventional IP (top) and SARIP (bottom). Ion rejection vs. hydrated radius is also presented in Supplementary Fig. 1 and Supplementary Table 1, which demonstrates a qualitatively similar comparison between the two PA membranes as shown here. The aqueous SDS concentration in SARIP is 2.1 mM. The rejection of different species was measured from NF experiments with the respective membranes using a cross-flow filtration cell with an operating pressure of 4 bar and a crossflow velocity of 2.9 cm s^−1^. Rejection data of each solute represents the average of three runs and error bar represents the standard deviation of three replicate measurements.

Here, we demonstrate the fabrication of an ultra-selective TFC-PA NF membrane capable of remarkable precise separation via alteration of the conventional IP process. In conventional IP, the NF membrane is formed via irreversible polymerization between piperazine (PIP) and trimesoyl chloride (TMC) at the water/hexane interface (Fig. 1a). To fabricate an ultra-selective NF membrane, we create a self-assembled network of amphiphiles at the water/hexane interface via the addition of sodium dodecyl sulfate (SDS). The well-organized and flexible interfacial network regulates the transport of PIP across the interface (Fig. 1b), forming a PA active layer with a highly uniform pore size distribution. Such an IP process in the presence of a self-assembled surfactant interfacial network is herein referred to as surfactant-assembly regulated interfacial polymerization (SARIP).

## Results

### Performance and properties of PA membrane formed via SARIP

The PA active layer formed via conventional IP has a heterogeneous pore (free volume) size distribution (Fig. 1c). The presence of SDS interfacial network in SARIP promotes the formation of a more uniform PA network (Fig. 1d), which in turn results in more precise differentiation between solutes of similar size. Although the addition of surfactants, including SDS, has been explored in numerous previous studies, they were added to promote the wetting of the support layer or the spreading of the water/oil interface; and the achievement of sub-1 Å level precision of solute-solute separation via surfactant addition has not been reported before^22–26^. SARIP provides a fundamentally different perspective regarding the impact of surfactants on interfacial diffusion of amine monomers and the overall IP process. While more quantitative evidence and mechanistic explanation will be provided later, the important impact of the interfacial regulation by the SDS network on the precision of solute sieving or selectivity of the PA layer is shown by comparing the rejection of different small solutes by TFC-PA membranes synthesized using IP and SARIP (Fig. 1e).

The TFC-PA membrane formed via conventional IP has a very wide range of rejection for solutes with Stokes radius, *r*_*s*_, between 2.5 and 5.0 Å (Fig. 1e, top). This wide distribution of rejection for species of similar size suggests that electrostatic and ion dehydration mechanisms contribute to ion rejection besides size-based sieving. While multivalent anions (e.g., SO_4_^2−^ and Fe(CN)_6_^3−^) are well rejected via Donnan exclusion by the negatively charged PA membrane formed via conventional IP, the rejection of multivalent cations is generally low and varies considerably with ion size (Supplementary Table 1). In contrast, the presence of interfacial SDS network in SARIP dramatically changes the solute separation behavior of the resulting PA membrane.

Notably, solute rejection becomes strongly dependent on the ion size and the measured rejection curve demonstrates a sharp, step-wise transition at *r*_*s* _~ 2.7 Å, separating monovalent and divalent cations with remarkable precision (Fig. 1e, bottom). Comparing the rejection curves for PA membranes prepared using IP and SARIP (Fig. 1e) shows that SARIP not only decreases the molecular weight cutoff, MWCO (i.e., the molecular weight corresponding to 90% rejection), but also reduces the range of the transition regime in the rejection curve, thus enabling differentiation of solutes with sub-1 Å selectivity (i.e., the rejections of two ions with a size difference smaller than 1 Å have a difference of at least 60%). For example, the rejections of Li^+^ (*r*_*s*_ = 2.4 Å) and Ba^2+^ (*r*_*s*_ = 2.9 Å) are 30% and 93%, respectively, with the PA membrane obtained using SARIP, whereas their rejections are very similar (19% and 17%) with the PA membrane derived using conventional IP. While Fig. 1e is based on Stokes radii to include both ions and neutral molecules, presenting the results for ions using hydrated radius reveals a similarly dramatic enhancement in the precision of ion-ion separation (Supplementary Fig. 1).

**Fig. 2:**
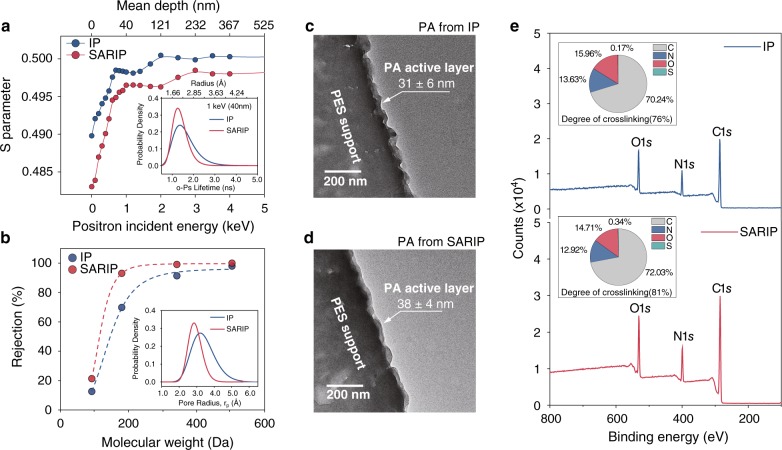
Properties of the PA active layers from IP and SARIP. **a** Evolution of *S* parameters for the PA active layers obtained using IP (blue circles and curve) and SARIP (red circles and curve). Inset: free-volume size distribution of PA active layers derived from the annihilation lifetime distribution of ortho-positronium (o-Ps) with incident energy of 1 keV. **b** Rejections of uncharged model solutes including raffinose, sucrose, glucose, and glycerol by TFC-PA membrane obtained from IP (blue circles and dashed curve) and SARIP (red circles and dashed curve). Inset: pore size distribution of PA active layers derived from rejection curves of uncharged solutes^28^. **c**, **d** TEM images of the cross-sections of the PA membranes fabricated using IP and SARIP, respectively. The translucent film in each image is the PA active layer and its thickness is analyzed using ImageJ at eight different locations. The reported PA layer thickness represented the average of eight measurements and the error bar represents the standard deviation of eight measurements. **e** XPS spectra and the corresponding elemental compositions (insets) of PA active layer for TFC-PA membranes fabricated using IP (top) and SARIP (bottom), respectively. The degree of crosslinking (shown in insets) is determined based on the ratio between elements O and N^29^.

The precise separation achieved by the TFC-PA membrane fabricated using SARIP is attributable to the more uniform pore size distribution of the SARIP-derived PA active layer as confirmed by both positron annihilation lifetime spectroscopy (PALS) (Fig. 2a) and characterization of pore size distribution using neutral solutes (Fig. 2b). PALS was used to probe the free volume distribution of the active layers^27^, which have a thickness between 30 and 40 nm as confirmed by cross-sectional transmission electron microscopy (TEM) images of the TFC-PA membranes (Fig. 2c, d). Specifically, the distribution of the *S* parameter of the TFC-PA membranes fabricated using the two different approaches suggests that SARIP yields pores that are both smaller and more uniform (Fig. 2a and Supplementary Table 2). These results from PALS are consistent with those obtained by fitting the rejection of neural organic molecules of different molecular weights (Fig. 2b). From both measurements, the sharpened pore size distribution of the PA layer resulting from SARIP still falls within the pore size distribution of PA layer obtained using conventional IP (Fig. 2a, b insets). Hence, the primary effect of SARIP was in sharpening the pore size distribution instead of merely shifting the pore size distribution to a smaller range.

While surface charge of the polymer plays a crucial role in Donnan exclusion—an important mechanism for ion rejection in NF^30,31^—streaming potential measurements reveal no discernable difference between the zeta potentials of the TFC-PA membranes formed via IP and SARIP (Supplementary Fig. 3). Further, X-ray photoelectron spectroscopy (XPS) analysis of the sulfur content in the PA active layers formed via the two fabrication methods suggests no integration of SDS molecule into the PA active layer, as long as the SDS concentration does not exceed the critical micelle concentration, CMC (Supplementary Figs. 4, 5 and Supplementary Tables 3, 4). The smaller pore size of the TFC-PA membrane fabricated using SARIP is likely attributable to the higher degree of crosslinking between the TMC and PIP in the PA structure (Supplementary Table 3). The elemental composition of the PA active layer as probed by XPS reveals that the degrees of crosslinking are 76% and 81% for the PA active layers formed via conventional IP and SARIP (Fig. 2e, Supplementary Table 3), respectively.

## Discussion

The formation of PA via IP is a complex, non-equilibrium, diffusion-reaction process. It is widely believed that PIP monomers in the aqueous phase diffuse across the water/hexane interface before they react with the TMC monomers in the hexane phase^15,16^. The trans-interface diffusion of PIP is the rate-limiting step because PIP only weakly partitions from water into hexane, whereas the reaction between PIP and TMC in hexane is fast. SARIP accelerates this rate-limiting PIP diffusion and also enhances the uniformity of the diffusional flux, which results in spatially more homogeneous polymerization that leads to a more uniform pore size distribution of the PA active layer.

**Fig. 3:**
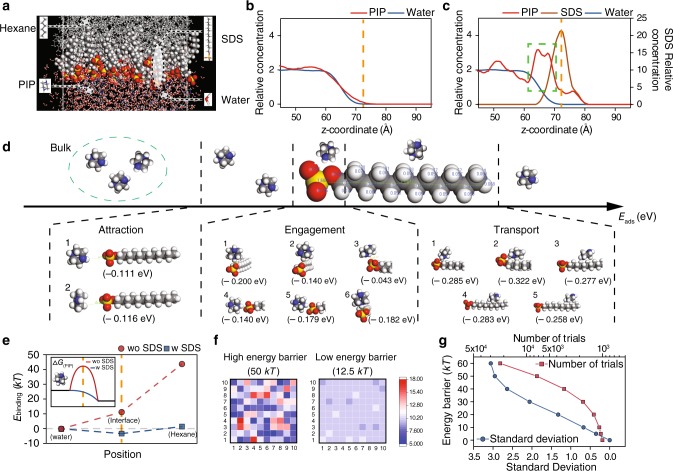
Facilitated trans-interface transport of PIP in SARIP. **a** A snapshot of the water/hexane interface in the MD simulation (Supplementary Fig. 6). **b** Relative abundance of PIP (red curve) and water (blue curve) across the interface (at ~70 Å, orange dashed line) in the absence of SDS. **c** Relative abundance of PIP (red curve), water (blue curve), and SDS (brown curve) across the interface (orange dashed line). SDS molecules accumulate at the interface and attract the PIP molecules. **d** DFT simulation of the potential energy for interaction between a PIP  molecule and an SDS molecule (with multiple configurations) at different interaction stages including attraction, engagement, and transport. **e** MD simulation of the binding free energy (*E*_binding_) with the PIP molecule at different locations (Supplementary Fig. 8). The MD simulations were performed with (blue squares) and without SDS (red circles). Inset: schematic illustration of how the Gibbs free energy barrier is reduced by the presence of SDS. **f** Monte Carlo simulation of particles with distributed energy passing through a 10 × 10 grid. The energy of the particles follows a Maxwell-Boltzmann distribution at 298 K. The color map represents the numbers of particles passing through different pixels (according to the scale bar) with an energy barrier of 50 kT (left) and 12.5 kT (right). **g** The total diffusion attempts (red squares and curve) and the standard deviation (blue circles and curve) of the spatial distribution of successful passages for 1000 particles passing through a 10 × 10 grid as functions of the free energy barrier.

To support our proposed mechanism, we performed molecular dynamics (MD) simulation of the diffusive transport of PIP monomers across the water/hexane interface in the presence of SDS (Fig. 3a and Supplementary Figs. 6–8). After the MD relaxation, we obtain an equilibrated structure, which forms a dynamic SDS network with an areal density of ~1.1 nm^−2^. This areal density is consistent with the experimentally measured surface excess concentration (Supplementary Table 5). The presence of SDS promotes the accumulation of PIP monomers near the water/hexane interface via electrostatic attraction between the negatively charged sulfonic group of SDS and the slightly positively charged PIP molecule (Fig. 3b, c and Supplementary Fig. 7).

Density function theory (DFT) simulation of the interaction between a PIP molecule and an SDS molecule in water shows that the mediated transport of a PIP molecule along the SDS chain is in general energetically favorable from the sulfonic head toward the dodecyl tail (Fig. 3d and Supplementary Figs. 9, 10). In addition, based on the binding free energy of a PIP molecule at multiple locations estimated from MD simulation results (without considering concentration gradient), the presence of SDS substantially reduces the binding energy penalty for a single PIP molecule to transport from the water phase to the hexane phase (Fig. 3e and Supplementary Fig. 10). At the level of an ensemble, the elevated PIP concentration at the water/hexane interface and the very fast reaction in the hexane phase induce a large concentration gradient and enhance the trans-interface transport of PIP. Overall, the interfacial network of SDS in SARIP facilitates the trans-interface transport of PIP from water to hexane by reducing the associated Gibbs free energy barrier.

Both the SDS-induced accumulation of PIP at the water/hexane interface, which creates a larger initial gradient for interfacial transport, and the SDS-modified energy landscape of trans-interface PIP transport, promote faster transport of PIP across the water/hexane interface and enhance the kinetics of the IP process. However, increasing the kinetics of IP is insufficient for forming a PA layer with more uniform pore sizes. For instance, increasing the kinetics of IP by using a higher PIP concentration can increase the degree of crosslinking and reduce MWCO (Supplementary Figs. 11, 12 and Supplementary Tables 6, 7), but it cannot result in the formation of more uniform pore size or precise differentiation of the rejections of monovalent and divalent ions (Supplementary Fig. 13 and Supplementary Table 7). More importantly, the SDS-facilitated trans-interface transport of PIP also results in spatially more uniform diffusion of PIP across the interface, which is essential for the formation of a PA membrane with a uniform pore size distribution. The relationship between energy barrier and flux uniformity can be semi-quantitatively illustrated by Monte Carlo (MC) simulations of the trans-interface transport of a group of particles with their kinetic energy following a Maxwell-Boltzmann distribution at a specific temperature. The underlying assumption is that, if the energy state of a particle exceeds a given energy barrier for trans-interface diffusion, that specific particle can successfully cross the interface. With this assumption, MC simulations of trans-interface diffusion events for 1000 particles across a 10 × 10 grid suggest that a lower energy barrier results in faster and more uniform flux across the interface (Fig. 3f, g). The total number of diffusion-attempts (trials) and the standard deviation of the numbers of particles passing through each grid opening decrease systematically as the energy barrier decreases (Fig. 3g), which suggests that reduced energy barrier does not only accelerate interfacial transport of PIP but also renders it more uniform. Additionally, the self-assembled SDS network at the water-hexane interface may also impose steric hindrance against the diffusion of newly formed PA fragments to water, which reduces the competing hydrolysis reaction and thereby also increases the degree of crosslinking. Therefore, both the enhancement of PIP diffusion and the suppression of undesirable hydrolysis synergistically lead to a higher degree of crosslinking (Fig. 2e) and a more homogeneous pore size distribution (Fig. 2a).

According to the mechanisms discussed above, not all surfactants can promote the formation of a more uniform PA active layer, even though they all reduce the interfacial tension between water and hexane (Supplementary Fig. 14) and promote better wetting of the support layer (Supplementary Fig. 15, Supplementary Table 8). For SARIP to achieve a more uniform PA layer, it requires the surfactants to be negatively charged so that they attract the positively charged PIP molecules. For example, SARIP with sodium dodecyl benzene sulfonate (SDBS), another anionic surfactant, shows qualitatively similar effect of enhancing pore size uniformity as imparted by SARIP with SDS (Table 1, Supplementary Figs. 16–18, and Supplementary Tables 9, 10). However, due to the steric hindrance of the benzene rings, SDBS has a lower interfacial packing density as compared to SDS (Supplementary Table 5), and is thus less effective in improving pore size uniformity. In contrast, adding other types of surfactants such as cetyltrimethylammonium bromide (CTAB, cationic) and sulfobetaine 3-14 (SB 3-14, zwitterionic) is ineffective in forming an active layer with more uniformly distributed pore size (Table 1, Supplementary Figs. 19–28, and Supplementary Tables 11–14). In particular, SARIP with CTAB undermines the selectivity of the PA active layer, resulting in larger and more heterogeneous pore size distribution (Table 1, Supplementary Figs. 19–22, Supplementary Tables 11, 12). The repulsion between the positively charged CTAB and PIP increases the energy barrier for trans-interface PIP diffusion, consequently leading to slower and more heterogeneous transport of PIP and hence less uniform pore size distribution. Lastly, sodium p-toluene sulfonate, which has a sulfonic group as SDS and SDBS but is nonetheless not a surfactant, shows no effect in improving the pore size distribution and the selectivity of the PA active layer (Table 1, Supplementary Figs. 29–31, and Supplementary Tables 15–18). This observation provides another indirect evidence that the enhanced selectivity of the PA layer formed via SARIP is attributable to the regulation of trans-interface PIP transport by the self-assembled surfactant network.

**Table 1:** Water permeance, rejection of selected salts, MWCO, and pore size distribution for different NF membranes.

Type^*^	Water permeance	Rejection (%)					MWCO	<*r*_*p*_>	*σ*_P_
	(L m^−2^ h^−1^ bar^−1^)	Na_2_SO_4_	MgSO_4_	MgCl_2_	CaCl_2_	NaCl	(Da)	*(*nm*)*	
*PIP (2.5% w v*^*−1*^*) with TMC*									
None^⊥^	12.6 ± 0.7	97.1 ± 0.5	95.3 ± 0.6	45.5 ± 0.8	24.7 ± 0.6	15 ± 1.1	274	0.334	1.219
SDS	17.1 ± 0.7	99.6 ± 0.9	98.2 ± 0.6	95.0 ± 0.6	93.0 ± 0.8	27.0 ± 0.7	208	0.31	1.177
SDBS	14.9 ± 0.8	98.8 ± 0.8	97.4 ± 0.7	82.0 ± 1.4	77.0 ± 1.1	21.3 ± 1.6	224	0.309	1.208
SB3-14	20.3 ± 0.9	99.1 ± 0.2	98.3 ± 0.8	89.6 ± 0.6	77.9 ± 0.3	20.9 ± 2.5	220	0.313	1.189
CTAB	26.2 ± 0.7	98.1 ± 0.3	90.9 ± 0.2	66.0 ± 1.3	32.0 ± 0.6	9.9 ± 0.7	302	0.362	1.194
TsNA^#^	14.9 ± 0.8	98.0 ± 0.2	95.4 ± 0.9	50.0 ± 1.2	29.2 ± 0.9	14.8 ± 1.0	255	0.326	1.212
*PEI (2.5% w v*^*−1*^*) with TMC*									
None	28.3 ± 0.9	39.7 ± 2.2	73.2 ± 2.6	88.6 ± 0.6	82.8 ± 1.3	30.7 ± 1.3	449	0.341	1.345
SDS	38.2 ± 0.9	62.5 ± 2.7	93.1 ± 3.1	95.8 ± 0.8	94.1 ± 0.9	46.1 ± 1.6	203	0.291	1.217

**Definitions**: MWCO molecular weight cutoff, determined using rejection curve of neutral solutes as in Fig. 2b, <*r*_p_> mean pore size, *σ*_p_ geometric standard deviation.

^⊥^No additive is added. This membrane serves as the baseline.

^*^Multiple concentrations have been tested for each additive (Supplementary Information) and the best performing results are reported here.

^#^All other additives are surfactants except for this case (sodium p-toluene sulfonate, or TsNA).

The effectiveness of SARIP in promoting more precise selectivity is not limited to the system of PIP and TMC as the reactive monomers. For example, it is well known that PA active layer formed from the reaction between polyethyleneimine (PEI) and TMC has a larger pore size distribution than that from PIP/TMC reaction^32^. Applying SARIP to the PEI/TMC system also resulted in smaller pore size and improved uniformity of pore size distribution as compared to the reference TFC-PA membrane formed via conventional IP with PEI and TMC (Table 1, Supplementary Figs. 32–34, and Supplementary Table 19).

**Fig. 4:**
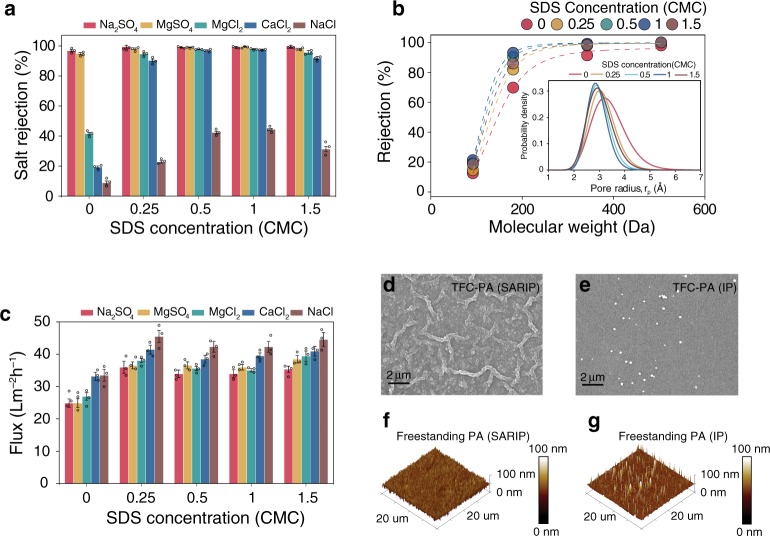
Concentration-dependent performance and active layer morphology. **a**–**c** Impacts of SDS concentration on the performance and properties of the TFC-PA membranes, including **a** rejection of various salts; **b** rejection of uncharged model solutes including raffinose, sucrose, glucose, and glycerol. Inset: pore size distribution of PA active layers derived from rejection curves of uncharged solutes (Supplementary Table 20); and **c** water flux. The hydraulic pressure was 4 bar and the salt concentration in the feed solution was 1000 ppm. **d**, **e** SEM images of the surface of TCF-PA membranes obtained using SARIP and conventional IP performed on a PES support, respectively; **f**, **g** AFM topography of free-standing PA films from SARIP and conventional IP, respectively. The free-standing films were fabricated without support and then transferred to silicon wafers. The error bars represent the standard deviation of data from three replicate measurements.

Because SDS self-assembly serves as a network of facilitators to enhance PIP transport across the water/hexane interface, increasing the interfacial density of such transport facilitators via increasing its bulk concentration is expected to enhance the positive effect of SARIP. Indeed, increasing the SDS concentration in the PIP solution up to 1 CMC enhances the salt rejection systematically (Fig. 4a) due to the smaller and more uniform size distribution of the resulting PA active layer (Fig. 4b and inset). However, using an SDS concentration of 1.5 CMC reverses the trend of the rejection improvement (Fig. 4a), likely due to the integration of SDS into the PA layer when micelles form. While the exact mechanism of micelle-assisted transport of SDS into the hexane phase remains to be elucidated, and S-2*p* peak was observed only in the XPS spectrum of the PA layer formed using SARIP with 1.5 CMC, but not in any XPS spectra of the PA layer formed using SARIP with 1.0 CMC and below (Supplementary Fig. 3). Further increasing the SDS concentration to 2 CMC leads to a more drastic reduction of salt rejection of the TFC-PA NF membrane (Supplementary Fig. 34).

The presence of SDS in the aqueous solution substantially enhances the membrane permanence, although the exact concentration of the SDS only has a comparatively minor impact (Fig. 4c). The permeance enhancement, even with a tightened pore sized distribution, is largely attributable to the larger specific surface area of the TFC-PA membranes formed via SARIP, as SARIP leads to the formation of a textured surface (Fig. 4d) compared to the presence of only sporadic protrusions on the surface of a TFC-PA membrane formed via conventional IP (Fig. 4e). The considerable enhancement of permeance by the formation of local texture has been extensively reported^19,20,33^. The formation of surface texture is not associated with the pore size distribution of the PA layer, as the texture characteristic length scale is two to three orders of magnitude larger than that of the pores. Rather, the emergence of the ridged structure is attributable to the enhanced wetting of the porous susbtrate^33^. Notably, performing SARIP at the water-hexane interface without the porous support yields a relatively smooth free-standing PA layer (Fig. 4f), In contrast, performing conventional IP (i.e., on a porous support) in the same setting still yields a PA layer with sporadic and large (*h* > 100 nm) protrusions (Fig. 4g).

Overall, SARIP represents a universally applicable process where the diffusive transport of amine monomers across the water/hexane interface is regulated by an organized and flexible network of anionic surfactants. Such a dynamic network promotes faster and more uniform flux of amine monomers across the water/hexane interface, which is necessary for the formation of a more homogeneous PA active layer. SARIP provides a fundamentally different perspective of the impact of surfactants on interfacial diffusion of amine monomers and the overall IP process. Notably, SARIP requires minimal alteration of the established techniques for fabricating conventional TFC-PA NF membranes, and can thus be readily implemented for the scalable fabrication of ultra-selective NF membranes for precise solute-solute separation.

## Methods

### Conventional IP

Conventional IP was performed using an aqueous solution of 0.25% w v^−1^ PIP and an n-hexane solution of 0.2% w v^−1^ TMC on a commercial polyethersulfone UF membrane (PES, NADIR UH050, MWCO 50,000 Da, Microdyn-Nadir, Germany) as the support layer. The PES UF membrane was first placed on a glass plate and then impregnated with PIP solution for 30 s. The glass plate was drained vertically, and a rubber roller was used to remove excess PIP solution from the UF membrane surface. Then the TMC solution was poured onto the membrane surface for another 30 s which resulted in the formation of a PA active layer over the PES membrane. The resulting TFC-PA membrane was immersed in n-hexane for 30 s to remove unreacted TMC, then heat-cured at 60 ^o^C for 30 min to increase the crosslinking degree of PA network. The membrane after heat curing was stored in water at 4 ^o^C to promote the hydrolysis of unreacted chloride groups in the PA network.

### Surfactant assembly regulated IP

The fabrication procedure in SARIP is similar to that in conventional IP as described above, except that surfactants are added to the PIP solution for forming an interfacial network. The concentration of each surfactant used during IP varies based on its critical micelle concentration (CMC). More details regarding the concentration of each surfactant can be found in Supporting Information.

### Characterization methods

A slow positron beam (VMSPB) was used to determine the free volume size and distributions of TFC-PA membrane from conventional IP and SARIP with SDS. This radioisotope beam used 50 mCi of ^22^Na as the positron source. Two positron annihilation spectroscopies were collected to explore the microstructure of the TFC-PA membrane: Doppler energy spectroscopy (DBES) and positron annihilation lifetime (PAL) spectroscopy. The DBES spectra were determined using PAS with a variable monoenergy slow positron beam (0–30 keV) and recorded using an HP Ge detector (EG&G Ortec). Surface elemental composition of PA active layers from conventional IP and SARIP was analyzed using a Thermal Fisher Scientific ESCALAB 250 Xi X-Ray photoelectron spectrometer. Cross-sectional TEM images of TFC-PA membranes prepared from conventional IP and SARIP were obtained using an FEI Tecnai G2 F20 S-twin 200 kV field-emission transmission electron microscope. Surface morphology of TFC-PA membranes from conventional IP and SARIP with SDS were characterized by a high-resolution Zeiss Merlin scanning electron microscope with GEMINI II column with an accelerating voltage of 3 kV. Samples were sputter-coated with gold (~5 nm thick) to inhibit the charging effect. The three-dimensional topography of freestanding PA films prepared from conventional IP and SARIP with SDS was measured with a Bruker Dimension Icon atomic force microscopy. Freestanding PA films were prepared using the same receipt as the fabrication of PA via conventional IP and SARIP, except that no PES support was used. The PA film formed at the water-hexane interface between PIP solution and hexane was transferred to a silicon wafer. The images were captured in tapping mode with RTESP probe (tip radius 8 nm, spring constant 40 Nm^−1^). A sampling resolution of at least 256 points per line and a speed of 0.1–1 Hz were used. The contact angle of the PIP aqueous solution with a variety of surfactants on the PES UF substrate was measured on an OCA20 instrument (Data-Physics, Germany) system at ambient temperature. The interfacial surface tension between n-hexane and PIP aqueous solution with and without surfactants was measured using the pendant drop method with OCA20 instrument (Data-Physics, Germany). The surface streaming potential of TFC-PA membranes prepared via conventional IP and SARIP with various surfactants was performed on an electro-kinetic Analyzer (SurPASS, Anton Paar, Ashland, VA) with an adjustable gap cell.

### Membrane performance evaluation

The performance of the fabricated NF membranes was tested using a system with three parallel stainless cross-flow filtration cells. Three different TFC-PA NF membranes were mounted into each of the three cells and tested at the same time. The active area of membranes in each cell was 7.1 cm^2^. The pure water permeability of PA NF membrane was measured using DI water before performing any NF experiments with feed solution containing solutes. The cross-flow velocity was 60 L h^−1^ and the applied pressure was 4 bar. The feed concentration of salts was 1000 ppm. The permeate flux was determined by measuring the weight change with respect to time, and ion selectivity was calculated based on the electrical conductivity of the feed and the permeate which was measured when stable permeating flux was achieved. Rejection of organic species (200 ppm) was also evaluated by measuring the total organic carbon (TOC) of the feed and permeate solutions using a TOC instrument (OI Analytical Aurora Model 1030). The rejections of organic species of different molecular weights are fitted to determine the MWCO and the pore size distribution of TFC-PA NF membranes. All measurements including solute rejection and membrane permeance were repeated three times with three different TFC-PA NF membranes fabricated from three individual IP processes.

The pure water permeance of TFC-PA membrane was calculated using Eq. (1):

1 $${\mathrm{PWP}} = \frac{{\Delta V}}{{S\Delta t\Delta P}}$$

where PWP is the pure water permeance of TFC-PA membrane (L m^−2^ h^−1^ bar^−1^), Δ*V* is the permeate water volume (L) collected over the period Δ*t* (h), *S* is the effective membrane area (m^2^), and Δ*P* was the applied pressure (bar), respectively.

The volumetric flux of water, *J* (L m^−2^ h^−1^ bar^−1^), was calculated using Eq. (2):

2 $$J = \frac{{\Delta V}}{{S\Delta t}}$$

The salt rejection, *R* (%), was calculated using Eq. (3):

3 $$R = \left( {1 - \frac{{c_{\mathrm{p}}}}{{c_{\mathrm{f}}}}} \right) \times 100{\mathrm{\% }}$$

where *R* is the salt rejection (%), c_p_ and c_f_ are the salt concentrations of the permeate and feed solution (ppm), respectively.

### Computational simulation

An Amorphous Cell module in Materials Studio was used to simulate the trans-interface diffusion of PIP from water to hexane with and with SDS. Two MD systems were constructed, one with a self-assembled SDS network at the water/hexane interface and the other without SDS. Both systems were comprised of the same numbers of H_2_O (5000), pip (100) and C_6_H_14_ (500) molecules in a lattice cell (50 × 50 × 140 Å^3^). In the MD model with SDS network, a total number of 36 SDS molecules were placed between water and hexane phases. After that, both MD systems were simulated for 20 ps with NVE thermodynamic ensemble at 298.0 K temperature. All the four reference energies (potential, non-bond, kinetic, and total energy) have reached the steady values after 10 ps. Meanwhile, the system temperature remained at the present value. The configurations at 15 ps in both MD systems were captured to analyze the population of pip molecules with and without the self-assembled SDS network. The relative concentrations of PIP, water and SDS molecules were shown in Fig. 3b, c.

To further explore the effect of the SDS dynamic network on the kinetics of PIP interfacial diffusion, we calculated the binding energy(*E*_binding_) of a PIP molecule to its surroundings at three sites: PIP bulk solution, water/hexane interface with and without SDS, and hexane.

4 $$E_{{\mathrm{binding}}} = E_{{\mathrm{X}} + {\mathrm{pip}}} - E_{\mathrm{X}} - E_{{\mathrm{pip}}}$$

where *E*_pip_ is the energy of one PIP molecule, *E*_X+pip_ is the total energy of the system including the PIP molecule and its surrounding, and *E*_X_ is the energy of the system without the PIP molecule, respectively.

To get the insight of how the interaction between a PIP molecule and an SDS molecule changed during the transport of PIP from water to hexane, we also performed a DFT simulation with Dmol modules in Material Studio. The molecular Frontier orbital of the PIP molecule and SDS molecule was calculated first in order to identify the population of the highest occupied molecular orbital (HOMO) and the lowest unoccupied molecular orbital (LUMO).

To simplify the DFT calculation, the transport of one PIP molecule along one SDS molecule was divided into three parts according to the location of PIP relative to SDS (Fig. 3d). Part 1 described the attraction between the SDS sulfate group and the PIP molecule in bulk solution (The distance between pip and SDS is around 5 Å); Part 2 was the engagement of the PIP molecule with the sulfate group; and in part 3, five different sites along the SDS alkane backbone were selected to discuss the change of interaction between PIP and SDS during transport. The adsorption energy (*E*_ads_) of PIP at each site was calculated using Eq. (5),

5 $$E_{{\mathrm{ads}}} = E_{ \ast + {\mathrm{pip}}} - E_ \ast - E_{{\mathrm{pip}}}$$

where *E*_pip_ was the energy of a single PIP molecule, $E_{ \ast + \mathrm{pip}}$ was the energy of the SDS molecule with the adsorption of PIP, and E_*_ was the corresponding energy of the SDS molecule without adsorption of PIP.

With the results from the MD and DFT simulations consistently showing that the presence of SDS may reduce the energy barrier for PIP diffusion across the water/hexane interface, we perform simplified MC simulations to illustrate why a lower energy barrier for diffusion can lead to more homogenous diffusive flux. In such an MC simulation, a group of generic particles (mimicking PIP molecules) attempt to pass a grid of cells (10 × 10 in this study) with a certain energy barrier, *ΔE*_B_. We assume that the intrinsic kinetic energy of these particles follows a Maxwell-Boltzmann distribution as expressed in using Eq. (6)

6 $${\mathrm{d}}N/N = \left( {\frac{m}{2{\uppi}k_{\mathrm{B}}T}} \right)^{1/2}\mathrm{exp}\left( { - \frac{mv^2}{2k_{\mathrm{B}}T}} \right)\mathrm{d}v$$

where d*N/N* is the fraction of PIP molecules moving at velocity *v* to *v* + d*v*, m is the mass of the PIP molecule, *k*_B_ is the Boltzmann constant and *T* is the absolute temperature. Therefore, the probability of one PIP molecule moving with a speed of v in three dimensions can be expressed as

7 $$p\left( v \right) = 4\pi \left( {\frac{m}{{2{\uppi}k_{\mathrm{B}}T}}} \right)^{3/2}v^2\mathrm{exp}\left( { - \frac{{mv^2}}{{2k_{\mathrm{B}}T}}} \right)$$

For each diffusion attempt across a cell in the grid, we randomly assign kinetic energy to a particle according to the Maxwell-Boltzmann distribution. If the energy of that particle is higher than the energy barrier (i.e., *ε*_*i*_ > Δ*E*_*B*_), the attempt is considered as successful and one additional particle is recorded as passing that specific cell. Otherwise, the attempt is considered as a failure and we move onto the next cell for the next diffusion attempt. Each cell has one diffusion attempt in each round (which comprises 100 attempts). The simulation continues until 1000 particles have successfully diffused across the 10 × 10 grids, resulting in an average of 10 particles per grid.

With the cumulative number of successful diffusions for each cell, we create a map of diffusion flux for the grid, with an example shown in Fig. 3f in the main text. The value of *E*_*B*_ has an impact on the distribution of diffusion flux, with a higher *E*_*B*_ leading to a more heterogeneous of diffusion flux and a lower *E*_*B*_ resulting in a more homogeneous diffusion flux. The heterogeneity can be quantified by calculating the standard deviation of the number of successful diffusions for different grids. We perform such simulations for a range of *E*_*B*_ to obtain the standard deviation and the total number of diffusion attempts (to generate 1000 successful diffusions) for each *E*_*B*_. The results presented in Fig. 3e in the main text show that a lower *E*_*B*_ leads to both faster diffusion (as quantified by fewer diffusion attempts) and a more homogeneous distribution of diffusion flux (as quantified by a lower standard deviation).

## Supplementary information

Supplementary Information

## Data Availability

All data are available in the manuscript or the supplementary materials from the authors on request.

## Code availability

The codes for simulations performed in this study (MD, DFT, and MC) are available upon request.
