# Supplementary material for: Polyamide nanofiltration membrane with highly uniform sub-nanometre pores for sub-1 Å precision separation
Source: Nat Commun. 2020 Apr 24;11:2015. doi: 10.1038/s41467-020-15771-2 (PMC7181833; doi:10.1038/s41467-020-15771-2)
Supplement: Supplementary file 1 — Supplementary Information [file 41467_2020_15771_MOESM1_ESM.pdf]

Supplementary Information

Polyamide Nanofiltration Membrane with Highly Uniform Sub-nanometre Pores for Sub-1Å Precision Separation

Yuanzhe Liang *et al*.

List of Content

Supplmentary Methods

Supplementary Figure 1-34

Supplementary Table 1-20

Supplementary References

1. Supplementary Methods

1.1. Chemicals and materials

Piperazine (PIP, 99%), trimesoyl chloride (TMC, >98%), sodium dodecyl sulfate (SDS, ⩾ 99.0%), hexadecyltrimethylammonium bromide (CTAB, BioXtra ⩾ 99%), 3-(N,N-Dimethylmytristylammonio) propane sulfonate (SB3-14, ⩾ 98%), sodium dodecylbenzene sulfonate (SDBS, technical grade), sodium p-toluene sulfonate (95%) poly(ethyleneimine) solution (average Mn ~ 10000, 50% in water), glycerol (⩾ 99%), anhydrous D-(+)-Glucose, sucrose (⩾99.5%), D-(+)-Raffinose pentahydrate (⩾ 98%), $K_3Fe(CN)_6$ (⩾ 99%), Na_2SO_4 (⩾ 99%), $MgSO_4$ (⩾ 99.5%), $ZnCl_2$ (99.99%), $CuCl_2$ (99.99%), $MgCl_2$ (⩾ 99.99%), $CoCl_2$ (99.9%), $CaCl_2$ (⩾ 97%), $NiCl_2$ (98%), $BaCl_2$ (99.99%), LiCl (⩾ 99%), NaCl (⩾ 99%), KCl (⩾ 99%), RbCl (⩾ 99%), CsCl (99.9%), $NaNO_3$ (⩾ 99%) were purchased from Sigma Aldrich (St. Louis, MO) and were all used as received. Anhydrous N-hexane and ethanol (HPLC) were purchased from Fisher Scientific. Polyester sulfone ultrafiltration (NADIR UH050, MWCO 50000 Da) membrane was purchased from Microdyn-Nadir (Germany).

1.2. Characterization methods

1.2.1. Positron annihilation spectroscopy (PAS)

A slow positron beam (VMSPB) was used to determine the free volume size and distributions of TFC-PA membrane from conventional IP and SARIP with SDS. This radioisotope beam used 50 mCi of ^{22}Na as the positron source. Two positron annihilation spectroscopies were collected to explore the microstructure of the TFC-PA membrane: Doppler energy spectroscopy (DBES) and positron annihilation lifetime (PAL) spectroscopy. The DBES spectra were determined using PAS with a variable monoenergy slow positron beam (0-30 keV) and recorded using an HP Ge detector (EG&G Ortec). Two parameters, R and S, were reported from the DBES measurement. The S parameter, which was from the o-Ps 2g pick-off annihilation in free volume, yielded information about the depth profile of the free volume (Å to nm) in the polyamide layer. Whereas the R parameter, defined as the 3g to 2g annihilation ratio, indicated the existence of large pores (nm to um) in which ortho-Positrinium (o-Ps) underwent 3g annihilation. The PAL spectra were analyzed

using the PATFIT to obtain the o-Ps lifetime τ_3 (1-5 ns), which was used to calculate the mean radius of the free volume (Å to nm) based on a semiempirical equation from a spherical-cavity model. The continuous o-Ps lifetime distribution was obtained from the MELT program to show the corresponding distribution of free volume in the PA network. Detailed descriptions of slow positron beam and PAS data analysis could be found elsewhere[1].

1.2.2. Scanning electron microscopy (SEM)

Surface morphology of TFC-PA membranes from conventional IP and SARIP with SDS were characterized by a high-resolution Zeiss Merlin scanning electron microscope with GEMINI II column with an accelerating voltage of 3 kV. Samples were sputter-coated with gold (~5 nm thick) to inhibit the charging effect.

1.2.3. X-ray photoelectron spectroscopy (XPS)

Surface elemental composition of polyamide active layers from conventional IP and SARIP was analyzed using a Thermal Fisher Scientific ESCALAB 250 Xi X-Ray photoelectron spectrometer. XPS specimens were prepared by carefully mounting polyamide films onto a silicon wafer. High-resolution scans in the carbon, nitrogen, oxygen, sulfur, and bromine regions were performed at 0.5 eV increments with a sweep time of 5000 s eV^{-1} and 25 energy sweeps for each region. XPS peak fitting was performed with XPSPEAK41 software.

1.2.4. Transmission electron microscopy (TEM)

Cross-sectional TEM images of TFC-PA membranes prepared from conventional IP and SARIP were obtained using an FEI Tecnai G2 F20 S-twin 200kV field-emission transmission electron microscope. TEM specimens were prepared by embedding TFC-PA membranes into epoxy resins, then ultrathin sections were prepared with a Leika EM UC7/FC7 microtome and carefully mounted onto lacey carbon support grids. The PA layer thickness was obtained by analyzing the TEM image using Image-J. Eight measurements were made at different locations along the PA layer. The reported PA layer thickness represented the average of eight measurements and the error bar represents the standard deviation of eight measurements.

1.2.5. Atomic force microscopy (AFM)

The three-dimensional topography of freestanding PA films from conventional IP and SARIP was measured with a Bruker Dimension Icon atomic force microscopy. Freestanding PA films

were prepared using the same receipt in section 2.1, except that no PES support was used. The PA film formed at the water-hexane interface between PIP solution and hexane were transferred to a silicon wafer. The images were captured in tapping mode with RTESP probe (tip radius 8 nm, spring constant 40 N m^{-1}). A sampling resolution of at least 256 points per line and a speed of 0.1 to 1 Hz were used.

1.2.6. Contact angle measurement

The contact angle of the PIP aqueous solution with a variety of surfactants on the PES UF substrate was measured on an OCA20 instrument (Data-Physics, Germany) system at ambient temperature. PES membranes were dried before measurement and mounted on glass slides. A drop of PIP aqueous solution (~3 ul) with different concentrations of surfactant was placed on the PES surface with a syringe. An optical image of the drop outline on the PES membrane surface was captured, and the corresponding water contact angle was calculated with a circle fitting method by drop shape analysis software.

1.2.7. Interfacial surface tension measurement

The interfacial surface tension between n-hexane and PIP aqueous solution with and without surfactants was measured using the pendant drop method with OCA20 instrument (Data-Physics, Germany). A transparent cubic container was filled with n-hexane, and one drop of PIP solution was generated from a syringe tip into hexane. An optical image of the drop hanging on the dosing needle was captured and the corresponding IFT value was calculated based on the Young-Laplace equation.

1.2.8. Streaming potential measurement

The surface streaming potential of TFC-PA membranes prepared via conventional IP and SARIP with various surfactants was performed on an electro-kinetic Analyzer (SurPASS, Anton Paar, Ashland, VA) with an adjustable gap cell. The streaming potential values were measured from pH 3 to 10 using 1 mM KCl solution as the background electrolyte at ambient temperature.

1.3. Membrane Fabrication

1.3.1. Preparation of Poly(piperazine-amide) nanofiltration membrane via conventional interfacial polymerization (IP)

Interfacial polymerization was first discovered in 1959 and remains state-of-the-art method for large-scale fabricating commercial polyamide nanofiltration (NF) and reverse osmosis (RO) thin-film-composite (TFC) membranes[2,3]. In this process, an ultrafiltration (UF) membrane (as the support layer) is wetted with an aqueous amine solution and then brought into contact with an immiscible organic solution containing acid chloride. Upon contact, amine molecules diffuse from the pores of the support membrane, across the water/oil interface, and react with acid chlorides in the oil phase to form the polyamide network.

In this study, conventional IP was performed using an aqueous solution of 0.25 % w v^{-1} piperazine and an n-hexane solution of 0.2 % w v^{-1} trimesoyl chloride on a commercial polyethersulfone (PES) UF membrane as the support layer. In the following discussion, all IP or SARIP recipes use this specified concentration of PIP and TMC unless otherwise noted. The PES UF membrane was first placed on a glass plate and then impregnated with PIP solution for 30 s. The glass plate was drained vertically, and a rubber roller was used to remove excess PIP solution from the UF membrane surface. Then the TMC solution was poured onto the membrane surface for another 30 s which resulted in the formation of a polyamide active layer over the PES membrane. The resulting TFC-PA membrane was immersed in n-hexane for 30 s to remove unreacted TMC, then heat-cured at 60 ^{o}C for 30 min to increase the crosslinking degree of polyamide network. The membrane after heat curing was stored in water at 4 ^{o}C to promote the hydrolysis of unreacted chloride groups in the polyamide network.

1.3.2. Poly(piperazine-amide) nanofiltration membrane from conventional IP with varying PIP concentrations

Conventional interfacial polymerization was conducted with varying PIP concentrations to study the effect of PIP concentration on the polyamide structure and performance. The PIP concentrations tested are 0.05 % w v^{-1}, 0.15 % w v^{-1}, 0.25 % w v^{-1} and 0.5 % w v^{-1}. The TMC concentration remained as 0.2 % w v^{-1} in all cases.

1.3.3. Poly(piperazine-amide) nanofiltration membrane from conventional IP (with sodium p-toluene sulfonate)

Sodium p-toluenesulfonate was added to PIP aqueous solution, as a comparison to sodium dodecyl sulfate, to study the effect of sulfate groups on the polyamide structure and performance. The concentrations of sodium p-toluenesulfonate correspond to the same molar concentration of sulfate groups as sodium dodecyl sulfate in water (4.1 mM Ts-Na vs. 0.5 CMC SDS, 8.2 mM Ts-Na vs. 1 CMC SDS, and 12.3 mM vs. 1.5 CMC SDS).

1.4. Preparation of polyamide nanofiltration membrane via surfactant assembly regulated interfacial polymerization (SARIP)

The fabrication procedure in SARIP is similar to that in conventional IP as described in 2.1, except that surfactants are added to the PIP solution for forming an interfacial network. More details regarding the addition of the surfactants are described below.

1.4.1. SARIP with the anionic surfactant, sodium dodecyl sulfate (SDS)

1.4.1.1. SARIP with SDS, PIP as amine

In this series of experiment, the SDS concentrations used were 2.05 mM (0.25 CMC), 4.1 mM (0.5 CMC), 8.2 mM (1 CMC) and 12.3 mM (1.5 CMC).

1.4.1.2. SARIP with SDS, polyethyleneimine (PEI) as amine

An aqueous solution of poly(ethyleneimine), or PEI, at a concentration of 0.25 % w v^{-1} was used in SARIP with an SDS concentration of 8.2 mM (1 CMC). The TMC concentration was 0.2 % w v^{-1}.

1.4.2. SARIP with the cationic surfactant, cetrimonium bromide (CTAB)

Cetrimonium bromide (CTAB), a cationic surfactant, as opposed to SDS as an anionic surfactant, was tested in SARIP to investigate the impact of charge of surfactant network on the properties of the PA layer. The concentrations of CTAB used in PIP solution were 0.23 mM (0.25 CMC), 0.46 mM (0.5 CMC), 0.92 mM (CMC), 1.84 mM (2 CMC), 2.76 mM (3 CMC), 4.6 mM (5 CMC), 7.36 mM (8 CMC), 9.2 mM (10 CMC) and 13.8 mM (15 CMC).

1.4.3. SARIP with the zwitterionic surfactant, 3-(N, N-Dimethylmyristylammonio) propane sulfonate (SB3-14)

A zwitterionic surfactant, 3-(N, N-Dimethylmyristylammonio) propane sulfonate (SB3-14) was also tested in SARIP for comparison with CTAB (cationic) and SDS (anionic) for their impacts on the properties of the resulting PA layer. The concentrations of SB3-14 used in PIP solution were 0.1 mM (0.25 CMC), 0.4 mM (1 CMC). 0.8 mM (2 CMC), 1.6 mM (4 CMC), 3.2 mM (8 CMC), 4.8 mM (12 CMC), 6.4 mM (16 CMC), 9.6 mM (24 CMC), 12.8 mM (32 CMC).

1.4.4. SARIP with sodium dodecylbenzene sulfonate (SDBS)

Another anionic surfactant, sodium dodecylbenzene sulfonate (SDBS), was investigated because SDBS chemical structure resembles that of SDS and should theoretically lead to qualitatively similar improvement of the properties of the PA layer according to the SARIP theory. The mechanism was explained in the following contents. The concentrations of SDBS in PIP solution were 0.6 mM (0.5 CMC), 1.2 mM (1 CMC) and 8.2 mM (1 CMC of SDS).

1.5. NF performance test of polyamide nanofiltration membranes from conventional IP and SARIP

The performance of the fabricated NF membranes was tested using a system with three parallel stainless cross-flow filtration cells. The active area of membranes in each cell was 7.1 cm^2. The pure water permeability of PA NF membrane was measured using DI water before performing any NF experiments with feed solution containing solutes. The cross-flow velocity was 60 L h^{-1} and the applied pressure was 4 bar. The feed concentration of salts was 1000 ppm. The permeate flux was determined by measuring the weight change with respect to time, and ion selectivity was calculated based on the electrical conductivity of the feed and the permeate which was measured when stable permeating flux was achieved. Rejection of organic species (200 ppm) was also evaluated by measuring the Total Organic Carbon (TOC) of the feed and permeate solutions using a TOC instrument (OI Analytical Aurora Model 1030). The rejections of organic species of different molecular weights are fitted to determine the molecular weight cutoff (MWCO) and the pore size distribution of TFC-PA NF membranes.

The pure water permeability of TFC-PA membrane was calculated by the following equation:

$$PWP = \frac{\Delta V}{S \Delta t \Delta P} \quad (1)$$

where PWP is the pure water permeability of TFC-PA membrane (L m^{-2} h^{-1} bar^{-1}), ΔV is the permeate water volume (L) collected over the period Δt (h), S is the effective membrane area (m^2), and ΔP was the applied pressure (bar), respectively.

The volumetric flux of water, J (L m^{-2} h^{-1} bar^{-1}), was calculated using the following equation:

$$J = \frac{\Delta V}{S \Delta t} \quad (2)$$

The salt rejection, R (%), was calculated using the following equation:

$$R = \left(1 - \frac{c_{\mathrm{p}}}{c_{\mathrm{f}}}\right) \times 100\% \quad (3)$$

where R is the salt rejection (%), c_{p} and c_{f} are the salt concentrations of the permeate and feed solution (ppm), respectively.

1.6. Determination of MWCO, pore size and pore size distribution of polyamide nanofiltration membranes from conventional IP and SARIP

The pore size of the polyamide network was determined by the rejection of a series of neutral organic compounds with increasing molecular weight. The neutral organic compounds tested in this study include glycerol (92 Da), glucose (180 Da), sucrose (342 Da) and raffinose (504 Da). The concentration of each organic species solution was 200 ppm and the applied pressure in the filtration experiments was 4 bar. The MWCO of TFC-PA membranes was defined as the molecular weight at which the rejection equals 90%. The pore size distribution curve is expressed as a probability density function (PDF) that was established based on the following assumption: (1) There is no steric or hydrodynamic interaction between these organic solutes and the membrane pores; (2) The mean pore size of the polyamide membrane equals the Stokes radius of the organic solute with a measured rejection of 50%; (3) The distribution of the membrane pore size is characterized by the geometric standard deviation of the PDF curve, which is the ratio between the Stokes radius with a rejection of 84.13% to that with a rejection of 50%[4].

$$\frac{dR(r_{\mathrm{p}})}{dr_{\mathrm{p}}} = \frac{1}{r_{\mathrm{p}} ln\sigma_{\mathrm{p}} \sqrt{2\pi}} \exp\left[-\frac{(lnr_{\mathrm{p}} - ln\mu_{\mathrm{p}})^2}{2(ln\sigma_{\mathrm{p}})^2}\right] \quad (4)$$

where μ_p is the mean pore size, σ_p is the geometric standard deviation of the PDF curve and r_p is the Stokes radius of the organic solute. The Stokes radii of these molecules correlate with their molecular weight[4]:

$$\ln(r_p) = -1.4962 + 0.4654 \ln(MW) \tag{5}$$

where MW is the molecular weight of each organic solute. Based on the above equation, the Stokes radii for glycerol, glucose, sucrose, and raffinose are 0.261, 0.359, 0.462, and 0.538 nm, respectively.

2. Supplementary data and figure

2.1. Separation mechanism in NF

Supplementary Table 1. Ionic radius, hydrated radius, hydration energy, and separation performance of ions investigated in this study. For consistency, data was collected from the same source[5,6]

	Ionic Radius (Å)	Stokes Radius (Å)	Hydrated Radius (Å)	Hydration energy (kcal mol^{-1})	Salt used in the test	Rejection (%)	
Cations						IP	SARIP
Zn^{2+}	0.74	3.49	4.30	467.3	$ZnCl_2$	61%	96%
Co^{2+}	0.72	3.35	4.23	457.7	$CoCl_2$	38%	96%
Mg^{2+}	0.65	3.47	4.23	437.4	$MgCl_2$	45%	95%
Cu^{2+}	0.72	3.25	4.19	480.4	$CuCl_2$	44%	94%
Ca^{2+}	0.99	3.10	4.12	359.7	$CaCl_2$	25%	93%
Ni^{2+}	0.70	2.92	4.04	473.2	$NiCl_2$	24%	93%
Ba^{2+}	1.35	2.90	4.04	298.8	$BaCl_2$	17%	93%
Li^{+}	0.60	2.38	3.82	113.5	LiCl	19%	30%
Na^{+}	0.95	1.84	3.58	87.2	NaCl	15%	27%
K^{+}	1.33	1.25	3.31	70.5	KCl	9.5%	20%
Rb^{+}	1.48	1.18	3.29	65.7	RbCl	13%	18%
Cs^{+}	1.69	1.19	3.29	59.8	CsCl	7.3%	16%
Anions							
$Fe(CN)_6^{3-}$	4.35	3.32	4.22	*596.3	$K_3Fe(CN)_6$	97%	100%
SO_4^{2-}	2.90	2.30	3.82	258.1	Na_2SO_4	96%	99%
NO_3^{-}	2.64	1.29	3.35	71.7	$NaNO_3$	8.4%	16%

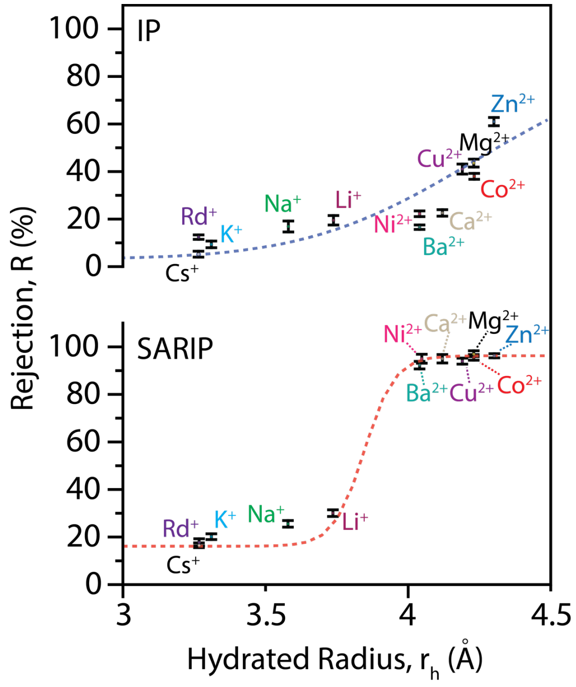

Supplementary Figure 1. Rejection of different ions as a function of hydrated radius for PA-TFC membranes fabricated using conventional IP (top) and SARIP (bottom). Hydrated radii of ions are presented in Supplementary Table 1. Rejection data of each solute represents the average of three runs and the error bars represent the standard deviation of the rejection from the three runs.

Separation mechanisms in NF include mainly steric (size sieving) and Donnan (charge) exclusion[7,8]. Solute molecules with a size that is larger than the membrane pore size are sterically blocked, while the transport of solutes with a size similar to that of the membrane pores may also be hindered. A membrane surface with a fixed charge repels ions with the same charge and attracts ions with the opposite charge. Because the poly(piperazine-amide) nanofiltration membrane has a net negative surface charge from the hydrolysis of unreacted TMC groups, it exhibits high rejection of SO_4^{2-} but relatively low rejection of Mg^{2+} and Ca^{2+}. The difference in selectivity for different cations with similar net charges and radii (Fig. 1d and Supplementary Figure 1) could be further explained based on the dehydration mechanism, i.e., an ion that approaches the membrane pore can strip and readjust its water shells temporarily in order to fit into the membrane pores. In general, smaller ionic size results in higher hydration energy, and ions with higher hydration energy are rejected more effectively by NF and RO membranes[9]. Ion dehydration, which is significant in NF because of the small pore sizes, offers an additional explanation for the differences in the rejection

of ions with similar charge and hydrated radii, e.g., Ni^{2+} (24%) and Ba^{2+} (17%), in the PA-TFC prepared from conventional IP.

2.2. Mean free-volume radius and free-volume radius distribution of PA from conventional IP and SARIP (with SDS) as assessed by Positron Annihilation Lifetime Spectroscopy (PALS)

Supplementary Table 2. Positron Lifetime results of polyamide active layer from conventional IP and SARIP (with SDS).

Sample	τ3 (ns)	Δτ3(ns)	I3 (%)	ΔI3 (%)	R (Å)	ΔR (Å)	ffv (%)	Δffv (%)
IP	1.434	0.039	12.155	0.417	2.266	0.044	1.066	0.099
SARIP (SDS)	1.297	0.043	14.831	0.434	2.095	0.054	1.028	0.110

τ3: o-Ps lifetime; I3: o-Ps density; R: mean free-volume radius; ffv: fractional free volume.

2.3. Surface streaming potential of TFC-PA membrane from conventional IP and SARIP (with SDS)

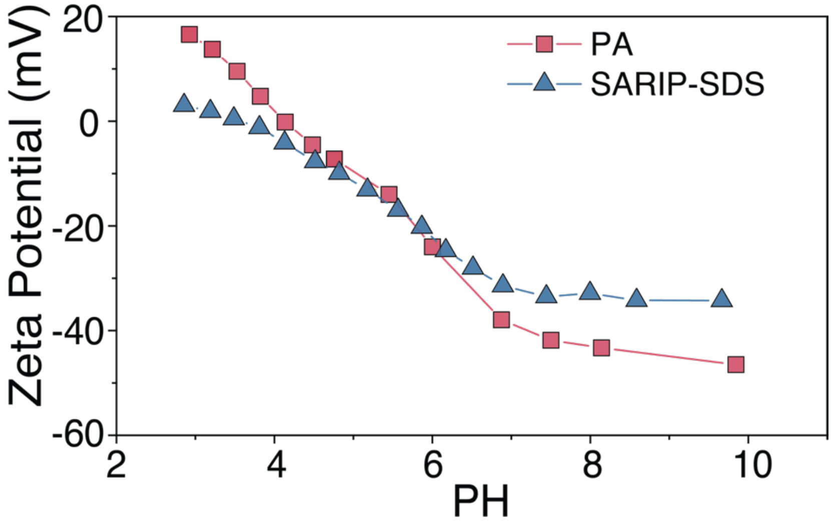

Supplementary Figure 2. Surface streaming potential of TFC-PA membranes from conventional IP and SARIP with SDS. (SDS Concentration: 1 CMC)

Both TFC-PA membranes from conventional IP and SARIP with SDS are negatively charged to a similar extent when pH is at 3 to 7. At a pH higher than 7, the surface of TFC-PA membrane prepared via conventional IP has a higher negative charge than that from SARIP because of the existence of more hydroxyl groups on the PA surface from the hydrolysis of unreacted TMC groups. It is in a good agreement with the XPS data in Supplementary Table 4.

2.4. XPS chemical characterization of the PA active layer from SARIP (with SDS)

2.4.1. XPS survey spectra of the PA active layer from conventional from SARIP (with SDS)

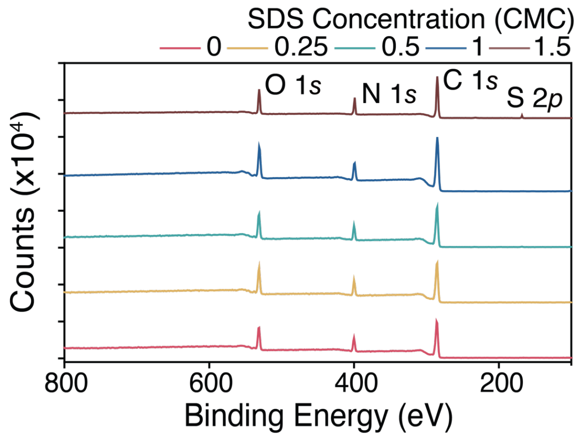

Supplementary Figure 3. XPS survey of polyamide active layer prepared via SARIP as a function of SDS concentration.

2.4.2. Calculation of degree of cross-linking of polyamide network

Supplementary Table 3. The elemental composition results and calculation of crosslinking degree of polyamide network obtained using SARIP (with SDS).

	C(%)	N(%)	O(%)	S(%)	Br(%)	Degree of crosslinking
SDS 0	70.24	13.63	15.96	0.17	0	0.76
SDS 0.25	70.38	13.64	15.69	0.29	0	0.79
SDS 0.5	71.03	13.48	15.28	0.2	0	0.81
SDS 1	72.03	12.92	14.71	0.34	0	0.81
SDS 1.5	70.78	12.81	14.9	1.51	0	0.77

The chemical compositions (C, N, O, S, Br) of polyamide active layer obtained using conventional IP and SARIP with SDS are listed in Supplementary Table 5. The degree of crosslinking for each system is calculated based on the following equation[10]:

$$\frac{O}{N} = \frac{3X + 4Y}{3X + 2Y} \qquad (6)$$

$$degree\ of\ crosslinking = \frac{X}{X + Y} \times 100\% \qquad (7)$$

2.4.3. High-resolution XPS spectra of poly(piperazine-amide) active layer from conventional IP and SARIP (with SDS)

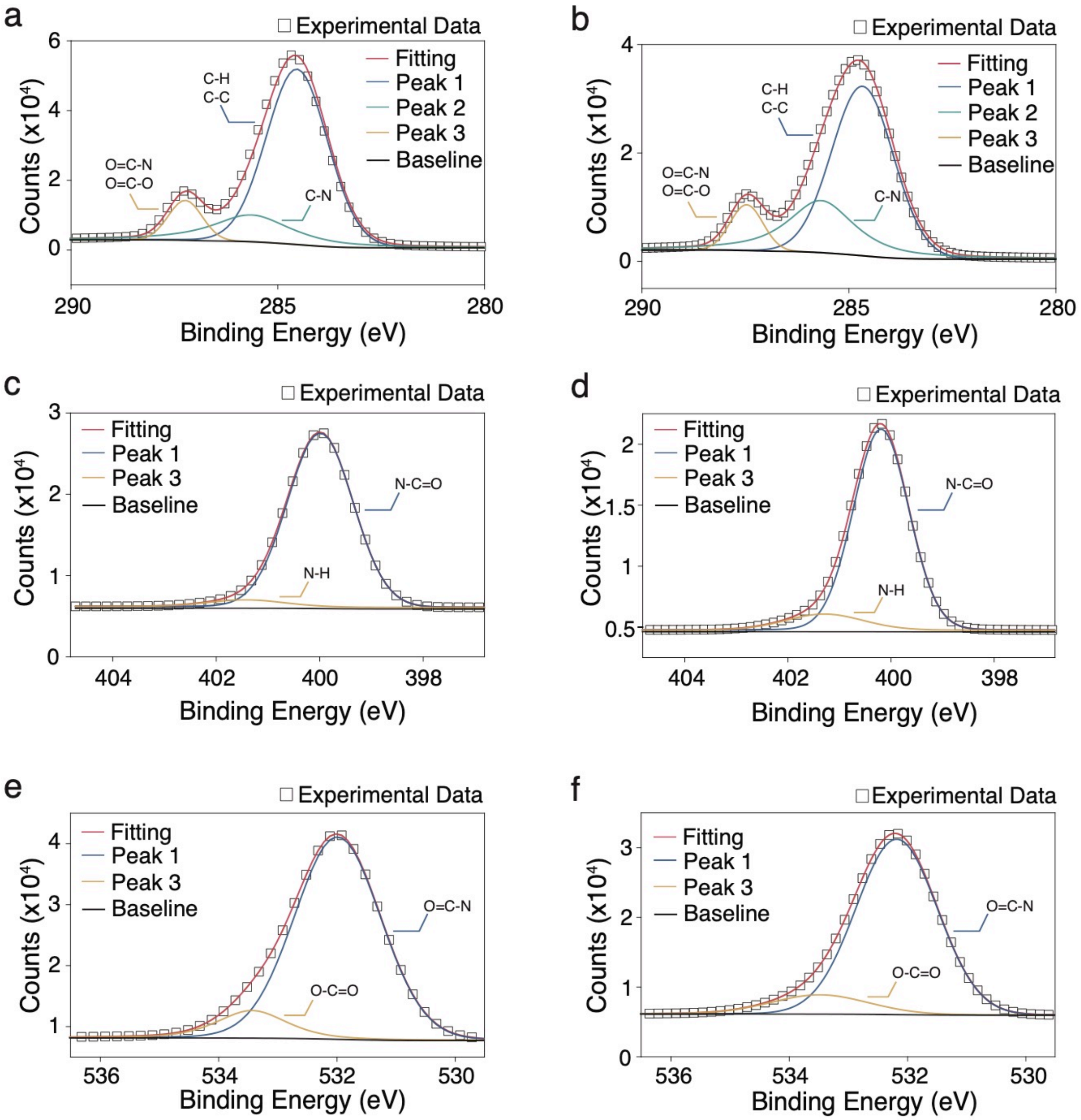

Supplementary Figure 4. High-resolution XPS spectra of polyamide active layer obtained using conventional IP and SARIP with SDS. (a and b) C 1*s* spectrum of conventional TFC-PA membrane and SARIP TFC-PA membrane. (c and d) N 1*s* spectrum of conventional TFC-PA membrane and SARIP TFC-PA membrane. (e and f) O 1*s* spectrum of conventional TFC-PA membrane and SARIP TFC-PA membrane. The chemical environment of carbon, nitrogen and oxygen was the

same in both TFC-PA membranes. Three C 1*s* peaks were detected, one at 284.6 eV (aliphatic/aromatic C-H or C-C bonds), the second one at 286 eV (C-N), and the other at 288 eV (amide O-C-N and carboxy O-C-O groups). Two N 1*s* signals were observed, peak one at 400 eV (N-C=O) and peak two at 401.7 eV (R-N-H). Both O 1*s* peaks at 532.0 eV (O=C-N) and 533.5 eV (O-C=O) were found in the TFC-PA from conventional IP and SARIP with SDS[11-14].

Supplementary Table 4. XPS results from the polyamide active layer from conventional IP and SARIP with SDS. Binding energies, plausible species and their content ratios were determined from the high-resolution C1*s*, N1*s*, and O1*s* XPS spectra.

Polyamide	C1*s*			N1*s*			O1*s*		
	Energy (eV)	Species	(%)	Energy (eV)	Species	(%)	Energy (eV)	Species	(%)
**IP**	284.6	C-H, C-C	66.8	400	N-C=O	92.2	532	O=C-N	88.1
	286	C-N	23.6						
	288	O-C=O, O-C=N	9.56	401.7	$-N^{+}H_2$	7.8	533.5	$O=C-O^{-}$ (H^{+})	11.9
**SARIP**	284.8	C-H, C-C	59.7	400	N-C=O	95.1	532	O=C-N	90
	286	C-N	28.7						
	288	O-C=O, O-C=N	11.6	401.7	$-N^{+}H_2$	4.9	533.5	$O=C-O^{-}$ (H^{+})	10

XPS C1*s*, N1*s* and O1*s* spectra of TFC-PA membrane prepared via conventional IP and from SARIP with SDS provided more detailed information of the chemical compositions of the PA active layer. The fractions of carboxylic acid groups and unreacted amine groups in the PA active layer from conventional IP were 11.9% and 7.8%, respectively; whereas the corresponding fractions of PA active layer from SARIP with SDS were 10% and 4.9%. This result indicated that the polyamide network prepared from SARIP with SDS contained more amide bonds and fewer unreacted PIP and TMC species compared to that from conventional IP.

2.5. Computational Simulations

2.5.1. Molecular Dynamics (MD) modeling of PIP diffusion with and without SDS

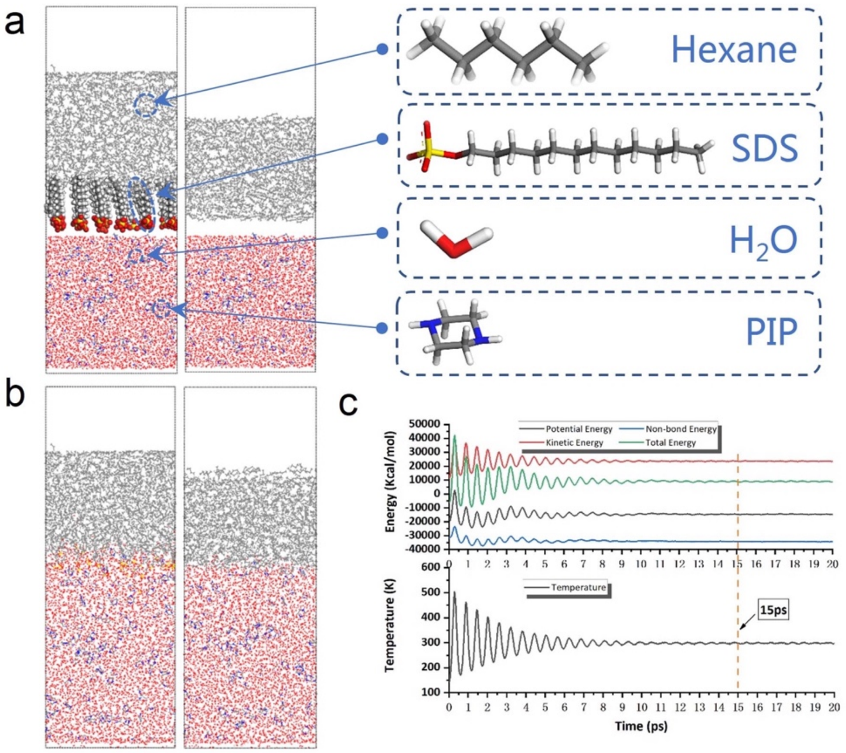

Supplementary Figure 5. (a) Initial MD models of PIP interfacial diffusion with and without self-assembled SDS network. (b) Two MD models at equilibrium. (c) Reference energy.

An Amorphous Cell module in Materials Studio was used to simulate the water-hexane interface. Two MD systems were constructed, one with a self-assembled SDS network at the water/hexane interface (Supplementary Figure 5 a,b left) and the other without SDS (Supplementary Figure 5 a,b right). Both systems were comprised of the same numbers of H_2O (5000), pip (100) and C_6H_{14} (500) molecules in a lattice cell (50×50×140 $Å^3$). In the MD model with SDS network, a total number of 36 SDS molecules were placed between water and hexane phases (Supplementary Figure 5a left). After that, both MD systems were simulated for 20 picoseconds with NVE thermodynamic ensemble at 298.0 K temperature. As shown in Fig S6 (c), all the four reference energies (potential, non-bond, kinetic, and total energy) have reached the steady values after 10ps. Meanwhile, the system temperature remained at the present value.

The configurations at 15ps in both MD systems were captured to analyze the population of pip molecules with and without the self-assembled SDS network (Supplementary Figure 6). The relative concentrations of PIP, water and SDS molecules were shown in Fig S8. The water/hexane interfaces in both MD systems were determined at 70 Å in the Z direction from two methods: in the MD system with SDS network, the interface could be informed from the crest of S atoms population where SDS molecules formed a dynamic self-assembled network with an interfacial areal density of 1.1 nm^{-1}; in the other system without SDS, the interface was determined by the minimum concentration of O atoms from water molecules. Due to the presence of the SDS network, a locally concentrated population of PIP molecules was observed at the water/hexane interface. A total number of 18 PIP molecules were found near the interface due to the formation of a dynamic SDS network vs. 8 PIP molecules near the interface without SDS. This result indicated that the presence of SDS promoted the accumulation of PIP monomers near the water/hexane interface.

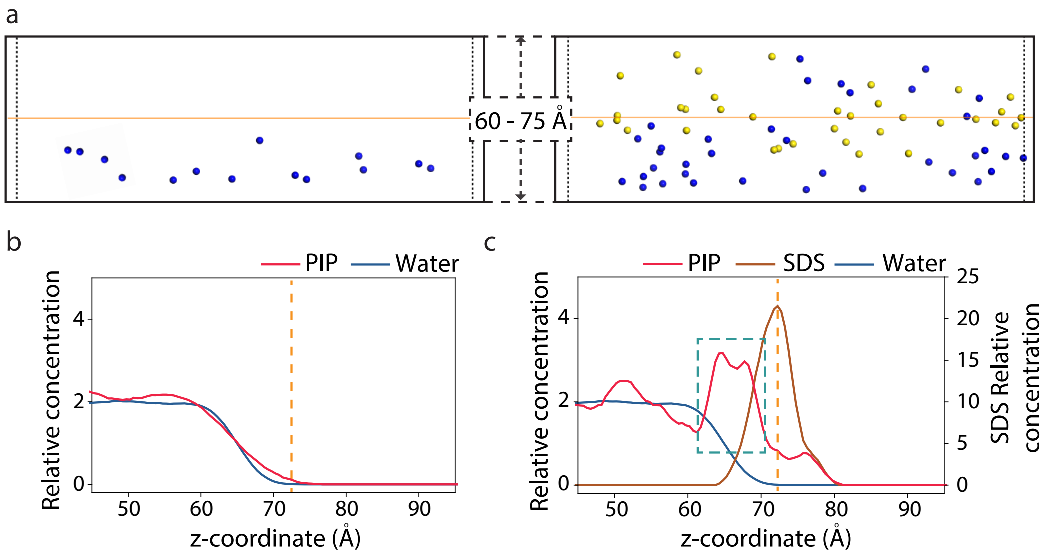

Supplementary Figure 6. (a) Distribution of PIP in the two MD models at equilibrium. Left: no SDS; Right: with SDS network. (b) Relative concentration of PIP (red) and water (blue) molecules close to the water/hexane interface in the absence of SDS. (c) Relative concentration of PIP (red), water (blue), and SDS (Orange) molecules close to the water/hexane interface.

To further explore the effect of the SDS dynamic network on the kinetics of PIP interfacial diffusion, we calculated the binding energy ($E_{binding}$) of a PIP molecule to its surroundings at three sites: PIP bulk solution (site 1), water/hexane interface with and without SDS (site 2), and hexane (site 3).

$$E_{binding} = E_{X+pip} - E_X - E_{pip} \quad (10)$$

where E_{pip} is the energy of one PIP molecule, E_{X+pip} is the total energy of the system including the PIP molecule and its surrounding, and E_X is the energy of the system without the PIP molecule, respectively.

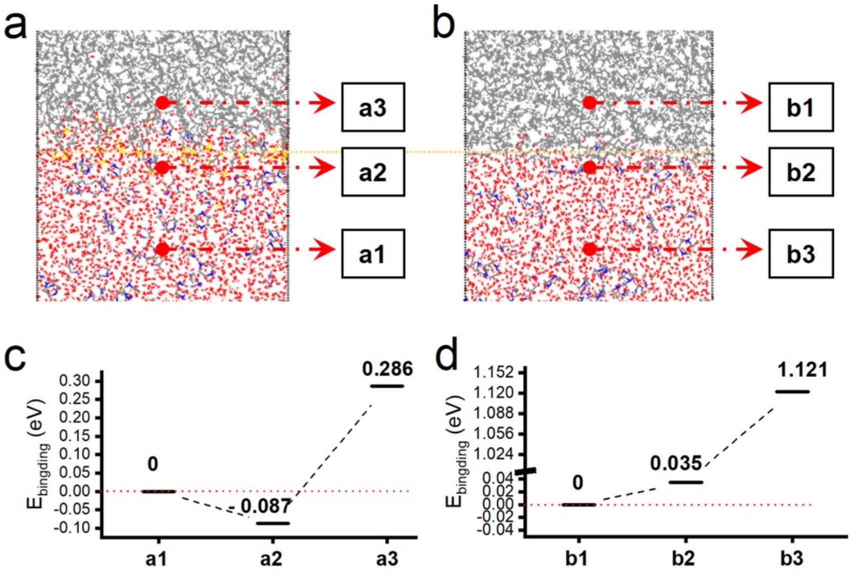

Supplementary Figure 7. Binding energy of one PIP molecule to its surrounding in the two MD systems, (a, c) with self-assembled network of SDS at water-hexane interface, (b, d) without SDS network, at three sites: a1, b1, water; a2, b2, water/hexane interface; a3, b3, hexane.

In the presence of an SDS dynamic network, $E_{binding}$ at the water/hexane interface (site a2), is calculated to be negative, which indicates that the transport of PIP molecules from bulk solution towards the interface is an energetically favorable process. This result is in good agreement with the locally concentrated population of PIP molecules near the interface. The further transport of a PIP molecule from the water/hexane interface into hexane needs to overcome an additional binding energy penalty of 0.29 eV with the presence of SDS network. Whereas, in the MD system without SDS network, the energy of PIP molecules at the interface is higher than that in the bulk solution, meaning that transport of PIP molecules to the interface is energetically unfavorable. The energy gain for a PIP to transport from the interface into hexane is 1.12 eV in the absence of SDS network, more than three times larger than that with SDS. Therefore, the formation of a self-assembled SDS

network at the water/hexane interface reduces the energy required for PIP to diffuse across the water/hexane interface.

While the results presented in Supplementary Figure 7 (c,d) seem to suggest that the diffusion of PIP from the aqueous to the hexane phase is both energetically unfavorable, the MD simulation performed to generate these results only consider the interaction between a PIP molecule and its medium but does not consider the effect of concentration gradient and the polymerization reaction in the hexane phase that depletes the PIP in hexane. The overall diffusion-reaction process is still energetically favorable if both the enthalpic (i.e., binding energy) and entropic contributions are considered. While the MD simulation of the entire diffusion-reaction process is technically highly challenging, the simulation of the binding energy presented in Supplementary Figure 7 is meant to semi-quantitatively show that the presence of the SDS will dramatically reduce the enthalpic gain of the diffusion and thus reduce the barrier of Gibbs free energy for the diffusion-reaction process.

2.5.2. Calculation of surface excess concentration (SEC)

The adsorption of surfactant molecules at the interface is driven by reducing the Gibbs free energy of the system[15-17]. Therefore, the concentration of surfactants at the interface is much higher than that of the bulk volume. Such difference of the concentration at the surface and any virtual interface in the bulk volume is called the surface excess concentration (SEC), Γ,

$$\Gamma = \Gamma_{\mathrm{I}} - \Gamma_{\mathrm{V}} \tag{8}$$

where the Γ_{I} is the interfacial concentration and Γ_{V} is the concentration at a virtual interface in the bulk solution.

The surface excess concentration is directly related to the interfacial surface tension (IFT) and can be calculated with the Gibbs adsorption isotherm equation[15]:

$$\Gamma = -\frac{1}{nRT}\frac{d\sigma}{dln(C)} \tag{9}$$

where the σ is the interfacial surface tension $(mN \cdot m)$, C is the surfactant molar concentration in the bulk $(mol\ L^{-1})$, T is the absolute temperature (K), R is the universal gas constant and n depends on the type of surfactant. (For ionic surfactants without extra electrolytes, like SDS, CTAB, etc., n equals 2)

Supplementary Table 5. Surface excess concentration results calculated from IFT data (Supplementary Figure 13).

Type of surfactant (CMC)	SDS (8.2 mM)	CTAB (0.92 mM)	SB3-14 (0.4 mM)	SDBS (1.2 mM)
SEC ($\times10^{-6}$ mol m^{-2})	1.82	1.64	1.47	1.50

2.5.3. Density Functional Theory (DFT) modeling of the interaction between PIP and SDS

To get the insight of how the interaction between a PIP molecule and an SDS molecule changed during the transport of PIP from water to hexane, we also performed a DFT simulation with Dmol modules in Material Studio. The molecular Frontier Orbital of the PIP molecule and SDS molecule was calculated first in order to identify the population of the Highest Occupied Molecular Orbital (HOMO) and the Lowest Unoccupied Molecular Orbital (LUMO). As shown in Fig S9 (a), in a PIP molecule, HOMO was located on the top area close to N and H atoms, and LUMO was located on the lateral area of the circle (Supplementary Figure 8b). In an SDS molecule, HOMO was located on the O atoms and LUMO was located in the middle of the alkane backbone (Supplementary Figure 8c,d).

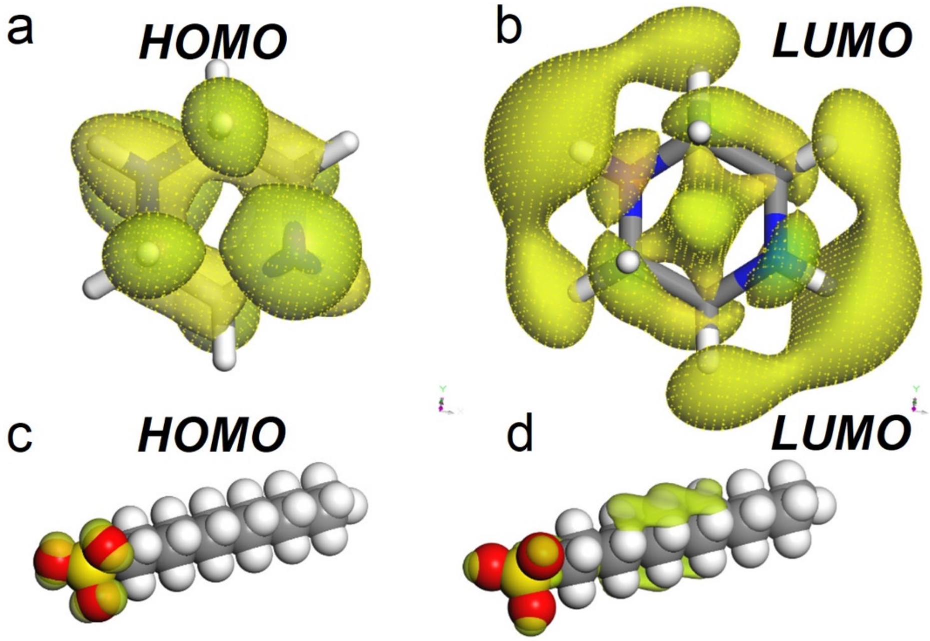

Supplementary Figure 8. Schematic illustration of HOMO and LUMO orbitals in the PIP and SDS molecule.

To simplify the DFT calculation, the transport of one PIP molecule along one SDS molecule was divided into three parts according to the location of PIP relative to SDS (Fig, S10). Part 1 described the attraction between the SDS sulfate group and the PIP molecule in bulk solution (The distance between pip and SDS is around 5Å); Part 2 was the engagement of the PIP molecule with the sulfate group; and in part 3, five different sites along the SDS alkane backbone were selected

to discuss the change of interaction between PIP and SDS during transport. The adsorption energy (E_{ads}) of PIP at each site was calculated by the following equation,

$$\mathrm{E_{ads}} = E_{*+\mathrm{pip}} - E_{*} - E_{\mathrm{pip}} \tag{11}$$

where E_{pip} was the energy of a single PIP molecule, $E_{*+\mathrm{pip}}$ was the energy of the SDS molecule with the adsorption of PIP, and E_{*} was the corresponding energy of the SDS molecule without adsorption of PIP.

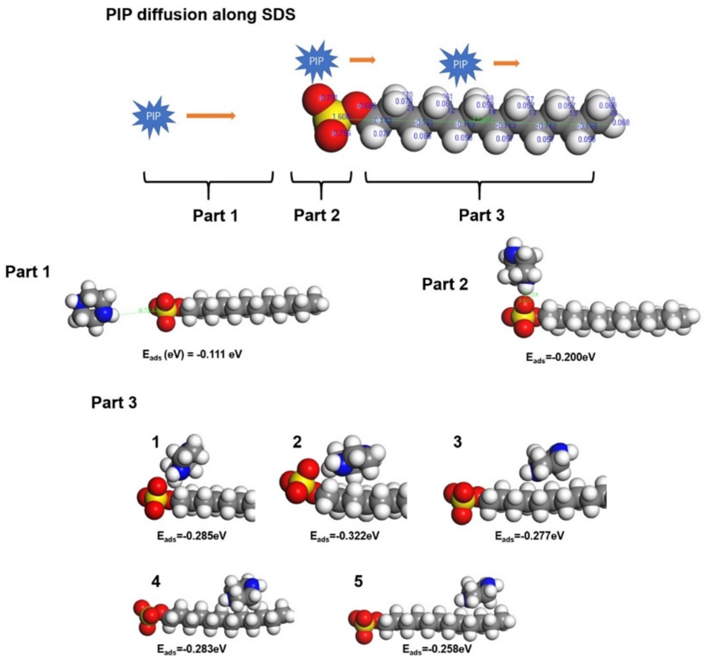

Supplementary Figure 9. Adsorption energy of a PIP molecule to a SDS molecule during the transport from water to hexane.

The negative adsorption energy in part 1 revealed the electrostatic attraction between the SDS sulfate group and PIP in bulk solution. The adsorption energy from part 1 to part 2 decreased because of the overlap of PIP's LUMO orbital and SDS's HOMO orbital. As the PIP molecule kept going along the SDS backbone, the adsorption energy started to increase when SDS's LUMO orbital triggered the overlap with PIP's HOMO orbital. This was in good agreement with the adsorption configuration. Combing the analysis from Molecular Frontier Orbital and the

calculation of adsorption energy by DFT, we can interpret the formation of an SDS self-assembled network promoted the diffusion of PIP from water to hexane.

2.6. Monte Carlo simulation of molecular diffusion across interface with different levels of energy barrier

With the results from the MD and DFT simulations consistently showing that the presence of SDS may reduce the energy barrier for PIP diffusion across the water/hexane interface, we perform simplified Monte Carlo (MC) simulations to illustrate why a lower energy barrier for diffusion can lead to more homogenous diffusive flux. In such an MC simulation, a group of generic particles (mimicking PIP molecules) attempt to pass a grid of cells (10 × 10 in this study) with a certain energy barrier, ΔE_B. We assume that the intrinsic kinetic energy of these particles follows a Maxwell-Boltzmann distribution as expressed in the following equation

$$\frac{dN}{N} = \left(\frac{m}{2\pi k_B T}\right)^{1/2} exp\left(-\frac{mv^2}{2k_B T}\right) dv \tag{12}$$

where dN/N is the fraction of PIP molecules moving at velocity v to $v + dv$, m is the mass of the PIP molecule, k_B is the Boltzmann constant and T is the absolute temperature. Therefore, the probability of one PIP molecule moving with a speed of v in three dimensions can be expressed as

$$p(v) = 4\pi \left(\frac{m}{2\pi k_B T}\right)^{3/2} v^2 exp\left(-\frac{mv^2}{2k_B T}\right) \tag{13}$$

For each "diffusion attempt" across a cell in the grid, we randomly assign kinetic energy to a particle according to the Maxwell-Boltzmann distribution. If the energy of that particle is higher than the energy barrier (i.e., $\varepsilon_i > \Delta E_B$), the attempt is considered as successful and one additional particle is recorded as passing that specific cell. Otherwise, the attempt is considered as a failure and we move onto the next cell for the next "diffusion attempt". Each cell has one diffusion attempt in each round (which comprises 100 attempts). The simulation continues until 1,000 particles have successfully "diffused" across the 10 × 10 grids, resulting in an average of 10 particles per grid.

With the cumulative number of successful diffusions for each cell, we create a map of "diffusion flux" for the grid, with an example shown in Fig. 3 (f) in the main text. The value of E_B has an impact on the distribution of diffusion flux, with a higher E_B leading to a more heterogeneous of diffusion flux and a lower E_B resulting in a more homogeneous diffusion flux. The heterogeneity can be quantified by calculating the standard deviation of the number of successful diffusions for different grids. We perform such simulations for a range of E_B to obtain the standard deviation and the total number of diffusion attempts (to generate 1,000 successful diffusions) for each E_B. The results presented in Fig. 3 (e) in the main text show that a lower E_B

leads to both faster diffusion (as quantified by fewer diffusion attempts) and a more homogeneous distribution of diffusion flux (as quantified by a lower standard deviation).

Notes: The MC simulations described above are highly simplified and are meant to illustrate qualitatively how reducing energy barrier leads to more uniformly distributed diffusion of molecules across an interface. The impact of the level of energy barrier on diffusion homogeneity may likely be even more significant due to the "positive feedback" mechanism. Specifically, when a PIP molecule successfully diffuses across an interface and reacts with TMC, a considerable amount of heat will be generated locally as the reaction of PIP and TMC is strongly exothermic. A large fraction of such released heat may propagate back to the water phase near the water/hexane interface (water is significantly more thermally conductive than hexane) and thereby increase the local temperature. The increased local temperature will result in higher thermal energy for the particles attempting to diffuse across and thus enhance the chance of successful subsequent diffusion. In short, a successful diffusion event will facilitate further successful diffusion near the same location, which is a positive feedback mechanism that tends to make the diffusion more heterogeneous. Accurately modeling this positive feedback mechanism is difficult and adds little to the already-highly-simplified MC simulation that is only qualitatively meaningful. We, therefore, do not attempt to perform such a simulation but only discuss the mechanism qualitatively.

2.7. Control experiment.

2.7.1. Poly(piperazine-amide) nanofiltration membrane from conventional IP

2.7.1.1. XPS chemical characterization of the PA active layer from conventional IP

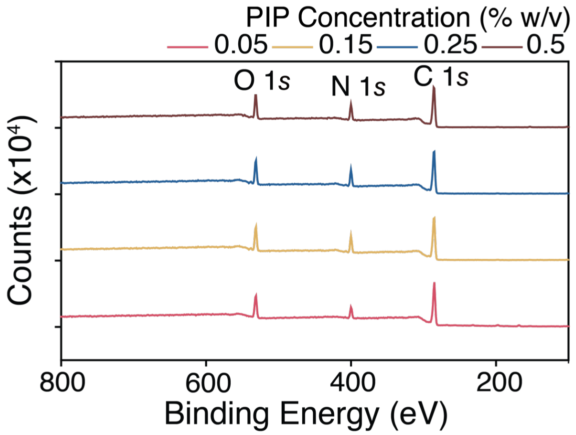

Supplementary Figure 10. XPS survey of polyamide active layer prepared via conventional IP as a function of PIP concentration.

2.7.1.2. Calculation of degree of cross-linking of polyamide network

Supplementary Table 6. The elemental composition results and calculation of crosslinking degree of polyamide network obtained using conventional IP.

	C(%)	N(%)	O(%)	S(%)	Br(%)	Degree of crosslinking
PIP 0.05%	73.05	10.58	15.4	0.97	0	0.44
PIP 0.15%	71.08	12.59	15.87	0.45	0	0.65
PIP 0.25%	70.24	13.63	15.96	0.17	0	0.76
PIP 0.5%	71.16	13.82	14.87	0.15	0	0.89

2.7.1.3. MWCO and pore size of TFC-PA membrane from conventional IP and SARIP (with SDS)

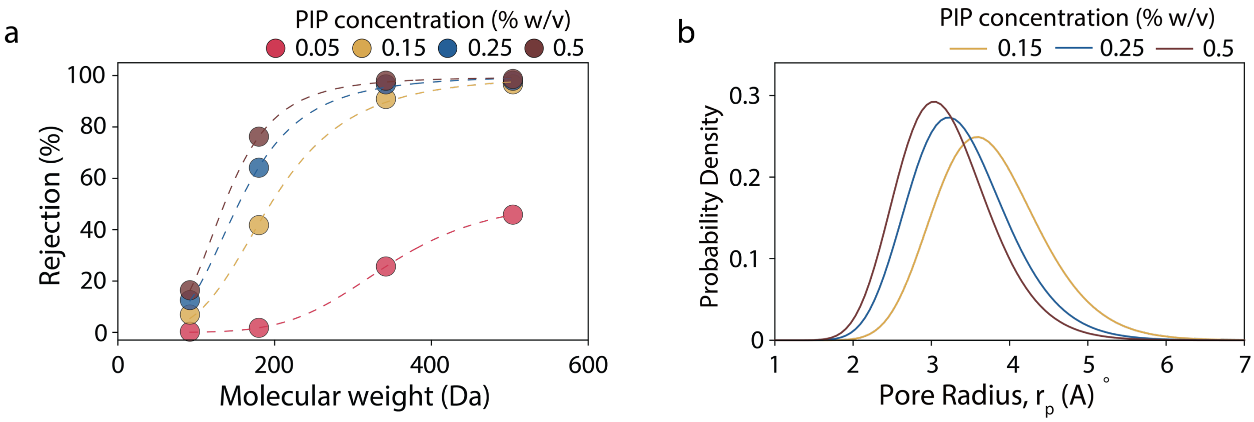

Supplementary Figure 11. (a) Rejection of neutral organic solutes of different MW by TFC-PA membranes fabricated using conventional IP with different PIP concentrations. (b) Pore size distribution estimated with data presented in (A) using Supplementary Equation 4.

Supplementary Table 7. Mean pore size, standard deviation and MWCO of TFC-PA membrane from conventional IP with different PIP concentrations.

PIP Concentration (% w v^{-1})	μ_p *(nm)*	σ_p	MWCO
0.05	N/A	N/A	N/A
0.15	0.371	1.200	346
0.25	0.334	1.219	274
0.5	0.314	1.207	232

2.7.1.4. Ion selectivity of TFC-PA membrane from conventional IP

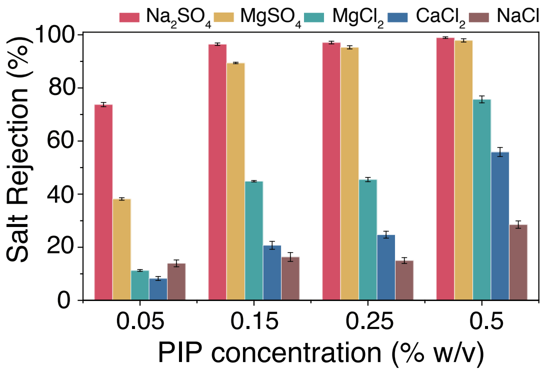

Supplementary Figure 12. Rejection of different solutes by TFC-PA membranes fabricated using conventional IP with different PIP concentrations.

Increasing the PIP concentration in IP increases the rejection of all ions systematically (Supplementary Figure 12), which is attributable to a higher degree of crosslinking of the PA layer (Supplementary Table 7) and smaller mean pore size (Supplementary Figure 11 a,c). However, even with the highest PIP concentration (0.5 % w v^{-1}, twice as the concentration used in SARIP), the rejection of Mg^{2+} and Ca^{2+} is still moderate as compared to that with TFC-PA membrane obtained using SARIP (with SDS) (Figure 4a). Therefore, even though one of the effects of SDS interfacial network is to enhance the interfacial concentration of PIP, the increase of PIP concentration at the interface is not the only mechanism for achieving a step-wise selectivity (e.g., Supplementary Figure 1b with 0.25 CMC of SDS) that is required for precise ion or molecular separation.

2.7.1.5. Interfacial Surface Tension (IFT) of piperazine aqueous solution and hexane with the addition of varieties of surfactants

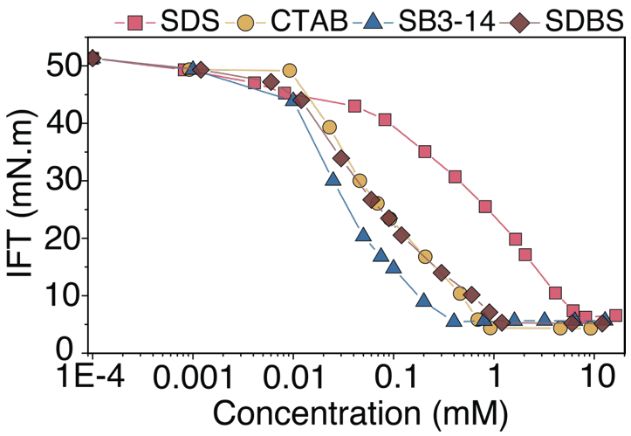

Supplementary Figure 13. IFT of hexane and piperazine aqueous solution with the addition of various concentrations of surfactants.

2.7.1.6. Wettability of piperazine aqueous solution on polyethersulfone ultrafiltration substrates with addition of various surfactants

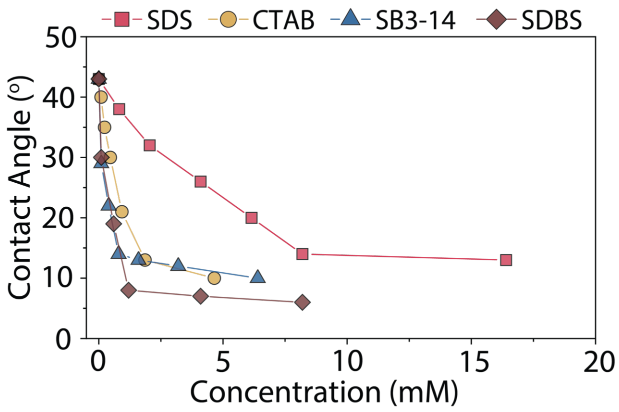

Supplementary Figure 14. Wettability of piperazine aqueous solution on polyethersulfone ultrafiltration substrate with addition of various surfactants. The addition of surfactants into the PIP aqueous solution lowers the water contact angle on the PES substrate. It indicates that the PIP solution could spread out better on the PES substrate, which leads to a complete water film and a more uniform distribution of PIP molecules on the substrate.

Supplementary Table 8. Water contact angle (WCA) of PIP solution on the PES substrate as a function of PIP concentration. Increasing PIP concentration did not affect the wettability of PIP solution on the PES substrate.

PIP Concentration (% w v^{-1})	0%	0.05%	0.15%	0.25%	0.5%
WCA	43 ± 2	37 ± 2	36 ± 2	36 ± 2	35 ± 2

2.7.2. Poly(piperazine-amide) nanofiltration membrane from SARIP (with SDBS)

2.7.2.1. Ion selectivity and permeate flux of TFC-PA membrane from SARIP (with SDBS)

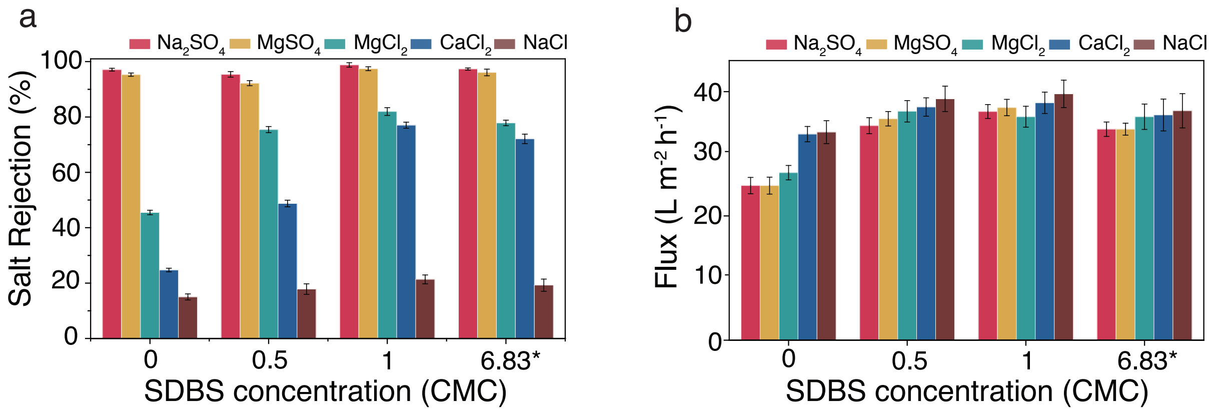

Supplementary Figure 15. (a) Rejection of different solutes by TFC-PA membrane fabricated using SARIP with different SDBS concentrations (b) permeate flux of TFC-PA membrane fabricated using SARIP with different SDBS concentrations. The flux was measured using different feed solution with a hydraulic pressure of 4 bar. *Note: The concentration of 6.83 CMC for SDBS corresponds to the same molar concentration (8.2 mM) as 1 CMC for SDS.

2.7.2.2. MWCO and pore size of TFC-PA membrane from SARIP (with SDBS)

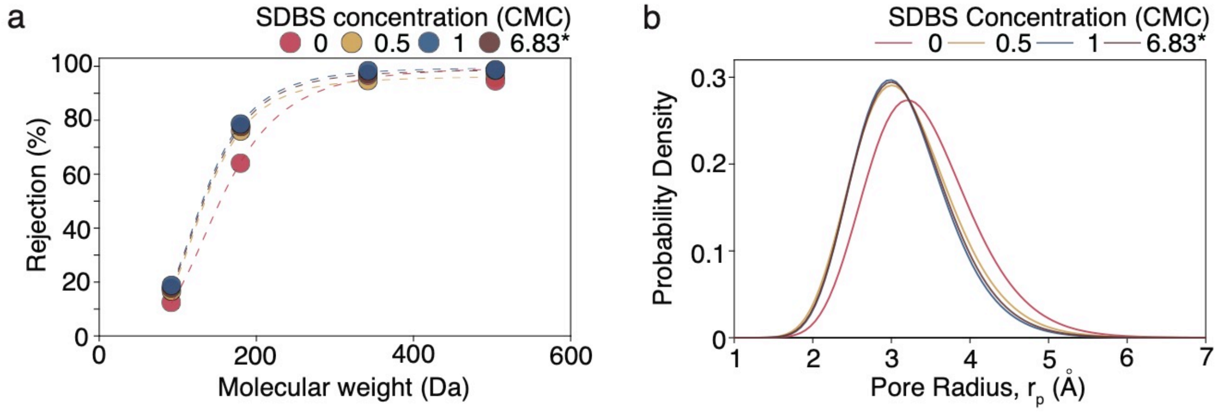

Supplementary Figure 16. (a) Rejection of neutral organic solutes of different MW by TFC-PA membrane fabricated using SARIP (with SDBS) with different SDBS concentrations. (b) Pore size distribution estimated with data presented in (a) using Supplementary equation 4. *Note: The concentration of 6.83 CMC for SDBS corresponds to the same molar concentration (8.2 mM) as 1 CMC for SDS.

Supplementary Table 9. Mean pore size, standard deviation and MWCO of TFC-PA from SARIP with different SDBS concentrations (corresponding to Supplementary Figure 16 a,b).

SDBS Concentration (CMC)	μ_p *(nm)*	σ_p	*MWCO (Da)*
0	0.334	1.219	274
0.5	0.313	1.224	246
1	0.309	1.208	224
6.83 (~ 8.2 mM, 1 CMC for SDS)	0.311	1.213	231

Similar to the effect of SDS on the formation of polyamide membranes, the pore size of TFC-PA from SARIP with SDBS became smaller than that of conventional TFC-PA. However, the relative low packing density of SDBS molecules (low surface excess concentration) leading to a less increase of PIP diffusion gradient, and the steric hindrance of the benzene ring on each SDBS molecule increased the diffusion difficulty. Therefore, the effect of SDBS on regulating the diffusion rate and uniformity of PIP was less effective than that of SDS. It could also be treated as a less reduction of diffusion activation energy in the Monte Carlo simulation.

2.7.2.3. XPS chemical characterization of the PA active layer from SARIP (with SDBS)

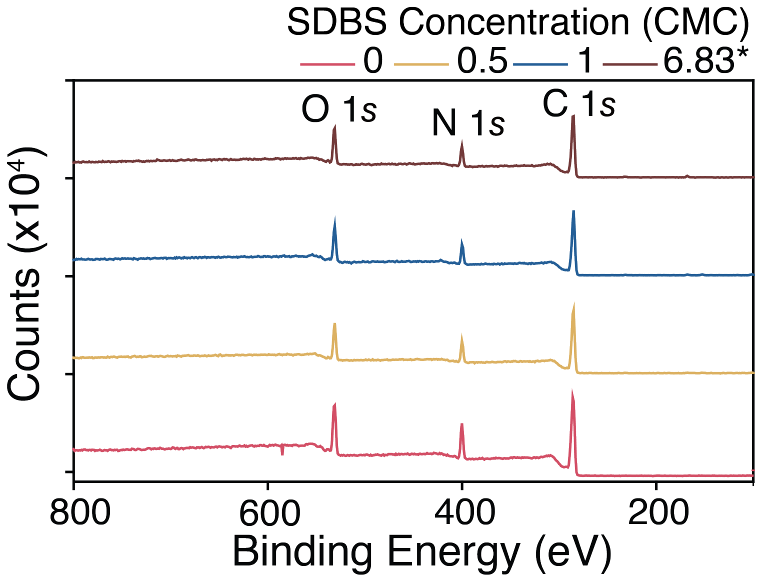

Supplementary Figure 17. XPS survey of polyamide active layer from SARIP as a function of SDBS concentration. *Note: The concentration of 6.83 CMC for SDBS corresponds to the same molar concentration (8.2 mM) as 1 CMC for SDS.

2.7.2.3.1. Calculation of degree of cross-linking of polyamide network

Supplementary Table 10. The elemental compositions and crosslinking degree of polyamide network from SARIP with SDBS.

SDBS concentration (CMC)	C(%)	N(%)	O(%)	S(%)	Br(%)	Degree of crosslinking
0	70.24	13.63	15.96	0.17	0	0.76
0.5	72.62	12.36	14.66	0.36	0	0.74
1	71.9	12.8	14.8	0.49	0	0.78
6.83 (~ 8.2 mM, 1 CMC for SDS)	71.41	12.87	14.98	0.73	0	0.77

2.7.3. Poly(piperazine-amide) nanofiltration membrane from SARIP (with CTAB)

2.7.3.1. Ion selectivity and permeate flux of TFC-PA membrane from SARIP (with CTAB)

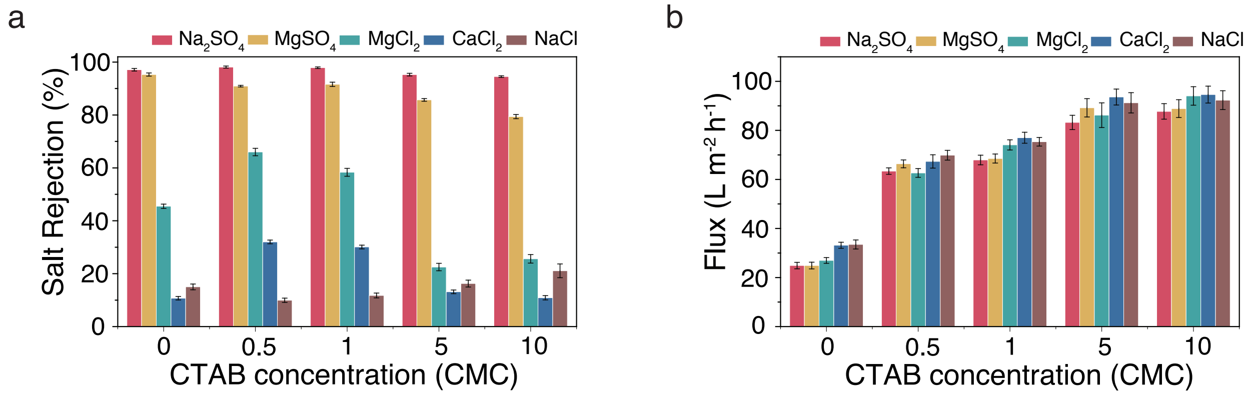

Supplementary Figure 18. (a) Rejection of different solutes by TFC-PA membrane fabricated using SARIP with different CTAB concentrations (b) permeate flux of TFC-PA membrane fabricated using SARIP with different CTAB concentrations. The flux was measured using different feed solution with a hydraulic pressure of 4 bar.

On the contrary to the function of SDS, the cationic surfactant, CTAB, exhibited the opposite effect on the ion selectivity performance of TFC-PA membranes from SARIP. In Supplementary Figure 18, an increase of CTAB concentration used in SARIP reduced the rejection of Na_2SO_4 and $MgSO_4$, and the rejection of $MgCl_2$, $CaCl_2$ and NaCl only increased a little at low concentrations and then dropped drastically. The initial increase of rejection of divalent ions and decreasing rejection of Na_2SO_4 and $MgSO_4$ could be caused by the change of surface charge density. In Supplementary Figure 26, the surface of TFC-PA from SARIP with CTAB was less negatively charged at the pH of 7. Such an increase in surface charge could be caused by the implementation of CTAB molecules into the polyamide network. Since the hydrophilic-lipophilic balance value of CTAB was 10 (HLB<10: water-insoluble and HLB>10: lipid insoluble), thus CTAB was a good O/W emulsifier. During the contact of aqueous PIP and organic phase TMC during interfacial polymerization, CTAB molecules were very likely to dissolve in hexane and competed with PIP to react with TMC. TFC-PA membrane prepared via SARIP with CTAB had a lower crosslinking degree and an increase of O/N ratio than that of the TFC-PA membrane prepared via conventional IP because each CTAB molecule had one-half of nitrogen atoms than a PIP molecule when they reacted with TMC. The presence of CTAB molecules in polyamide network added more free

volumes and reduced the surface negative charge. Overall this caused the reduction of rejection of $MgSO_4$ and Na_2SO_4 and higher selectivity of $MgCl_2$ and $CaCl_2$ at a low concentration of CTAB. As the concentration of CTAB increased, the more CTAB molecules reacted with TMC in hexane, causing more defects in the polyamide network, leading to the increasing reduction of ion selectivity of the polyamide nanofiltration membrane.

2.7.3.2. MWCO and pore size of TFC-PA membrane from SARIP (with CTAB)

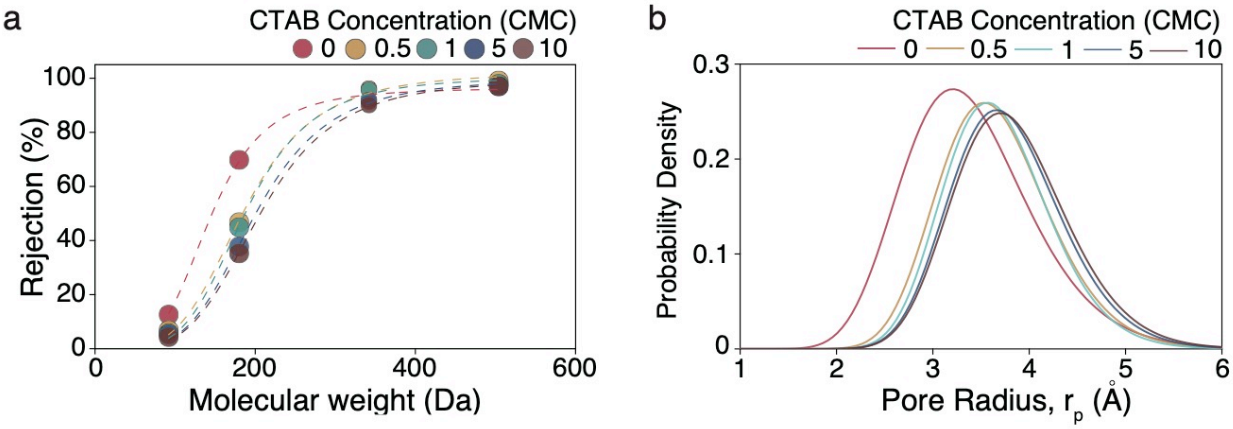

Supplementary Figure 19. (a) Rejection of neutral organic solutes of different MW by TFC-PA membrane fabricated using SARIP (with CTAB) with different CTAB concentrations. (b) Pore size distribution estimated with data presented in (a) using Supplementary Equation 4.

Supplementary Table 11. Mean pore size, standard deviation and MWCO of TFC-PA membrane from SARIP with different CTAB concentrations (corresponding to Supplementary Figure 19 a,b).

CTAB concentration (CMC)	μ_p *(nm)*	σ_p	*MWCO (Da)*
0	0.334	1.219	274
0.5	0.362	1.194	303
1	0.365	1.201	301
5	0.375	1.206	335
10	0.379	1.208	347

2.7.3.3. **XPS chemical characterization of PA active layer from SARIP (with CTAB)**

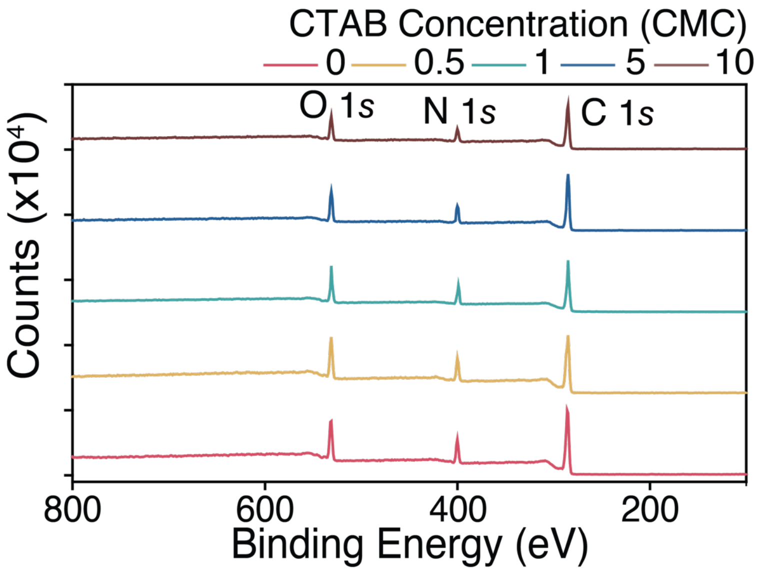

Supplementary Figure 20. XPS survey of polyamide active layer from SARIP as a function of CTAB concentration.

2.7.3.3.1. **Calculation of degree of cross-linking of polyamide network**

Supplementary Table 12. The elemental composition and crosslinking degree of polyamide network from SARIP with CTAB. The Bromine composition increased with the increase of CTAB concentration used in the SARIP.

CTAB Concentration (CMC)	C(%)	N(%)	O(%)	S(%)	Br(%)	Degree of crosslinking
0.5	71.46	12.51	15.88	0.12	0.03	0.64
1	72.5	12.08	15.12	0.23	0.07	0.66
5	75.15	10.44	14.1	0.26	0.05	0.55
10	74.7	10.62	14.28	0.31	0.1	0.56

2.7.3.4. Cross-sectional TEM images of TFC-PA membranes from SARIP (with CTAB)

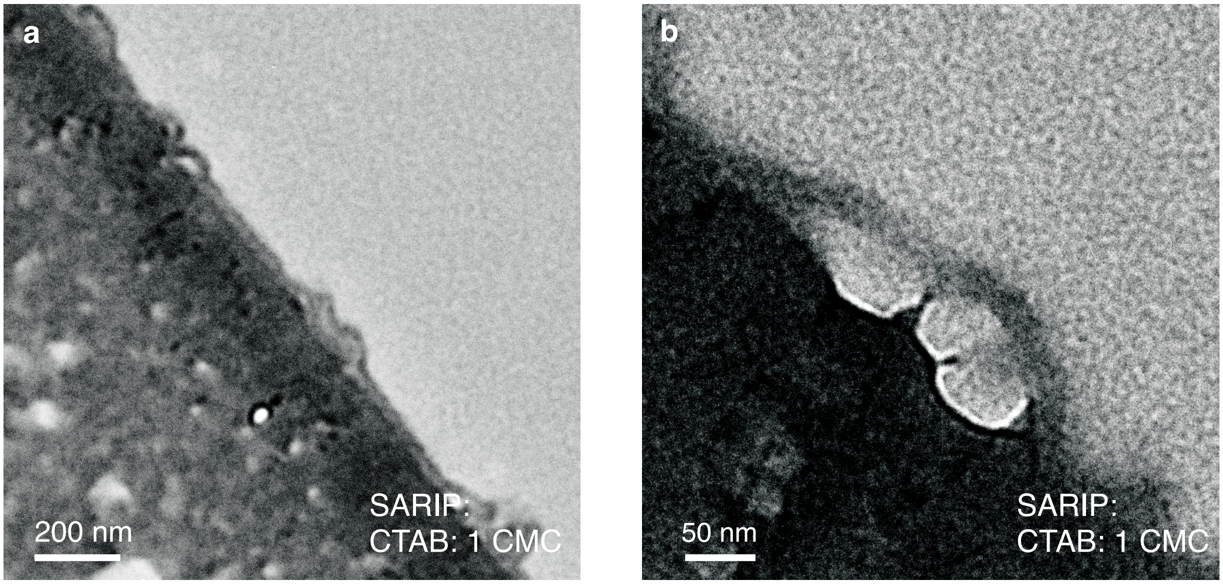

Supplementary Figure 21. Cross-sectional TEM images of TFC-PA membrane obtained using SARIP with CTAB (concentration: 1 CMC). Large voids were observed in the PA active layer, which contributed to the increase of membrane flux.

2.7.4. Poly(piperazine-amide) nanofiltration membrane from SARIP (with SB3-14)

2.7.4.1. Ion selectivity and permeate flux of TFC-PA membrane from SARIP (with SB3-14)

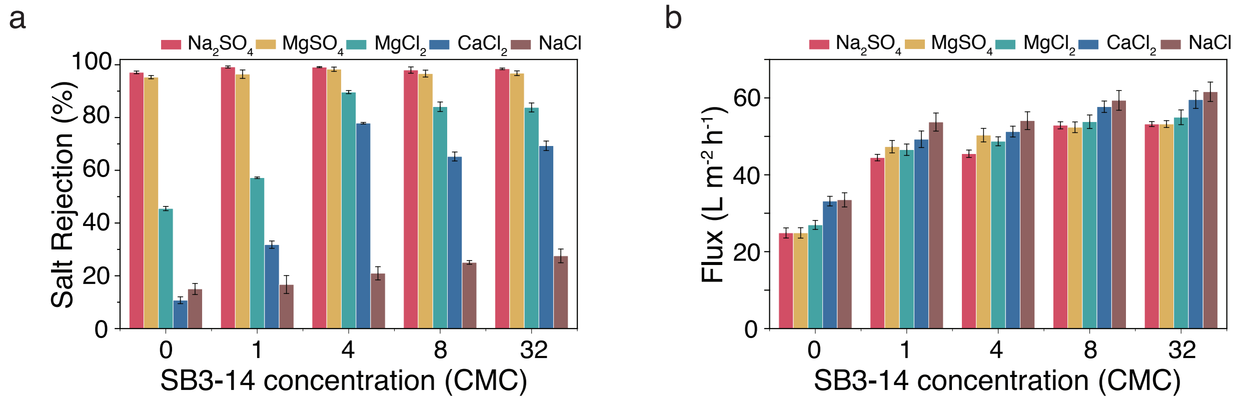

Supplementary Figure 22. (a) Rejection of different solutes by TFC-PA membrane fabricated using SARIP with different SB3-14 concentrations (b) permeate flux of TFC-PA membrane fabricated using SARIP with different SB3-14 concentrations. The flux was measured using different feed solution with a hydraulic pressure of 4 bar.

A zwitterionic surfactant was also explored to strengthen the gap between the cationic and anionic surfactant. Sulfobetaine 3-14 was chosen because it had a cationic tertiary amine group like CTAB and an anionic sulfate group like SDS, and its unique molecular structure did not form an intramolecular bond between its cationic group and anionic group. The HLB value of SB3-14 was 48.45 calculated with Davie's method, which was larger than the HLB value of SDS (40). Therefore, it was unlikely for SB3-14 to dissolve in hexane and interrupt the reaction between PIP and TMC (No sulfate element was detected in the XPS data). The general trend of the ion rejection of TFC-PA from SARIP with SB3-14 resembled the result of TFC-PA from SARIP with SDS. The pore size of TFC-PA prepared from SARIP with SB3-14 was slightly reduced than that of TFC-PA prepared from conventional IP as well as the pore size distribution, which contributed to the increase of ion selectivity.

2.7.4.2. MWCO and pore size of TFC-PA membrane from SARIP (with SB3-14)

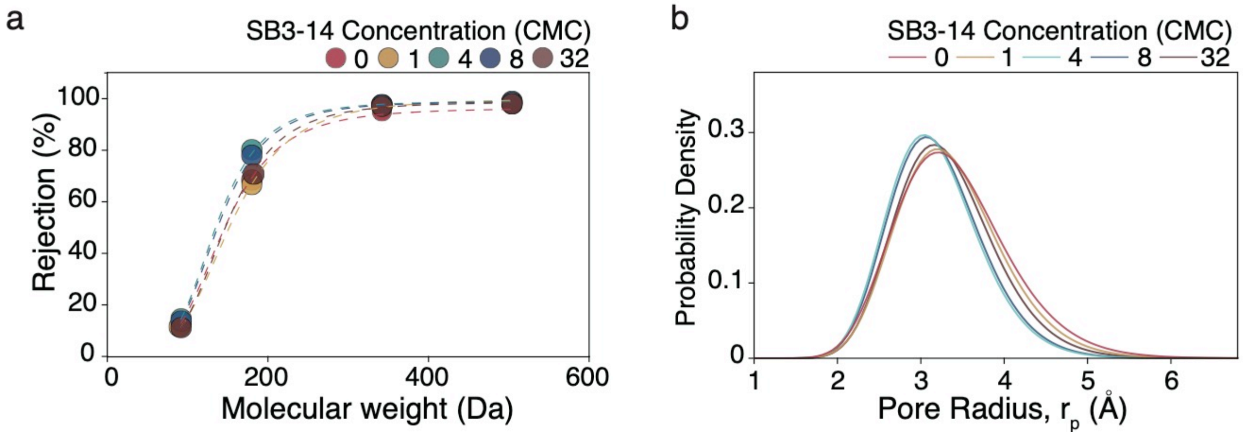

Supplementary Figure 23. (a) Rejection of neutral organic solutes of different MW by TFC-PA membranes fabricated using SARIP (with SB3-14) with different SB3-14 concentrations. (b) Pore size distribution estimated with data presented in (a) using Supplementary Equation 4.

Supplementary Table 13. Mean pore size, standard deviation and MWCO of TFC-PA membrane from SARIP as a function of SB3-14 concentration. (corresponding to Supplementary Figure 23 a,b)

SB3-14 concentration (CMC)	μ_p (nm)	σ_p	MWCO (Da)
0	0.334	1.219	274
1	0.331	1.204	259
4	0.313	1.189	220
8	0.316	1.190	226
32	0.326	1.196	246

The pore size of TFC-PA membrane from SARIP with SB3-14 decreased compared to TFC-PA membrane from conventional IP, so was the pore size distribution. The effect of SB3-14 on the formation of polyamide during interfacial polymerization resembled the function of SDS molecules, but less effectively. A monolayer of self-assembled SB3-14 network at the water/hexane interface attracted the PIP molecules to their hydrophilic ends, leading to an increase of PIP diffusion gradient. However, transport of PIP molecules along the SB3-14 chain was arduous because of the electrostatic repulsion from the cationic tertiary amine group. Such a trade-

off effect could be treated as a less pronounced reduction of the diffusion energy barrier compared with the effect of SDS self-assembly network. Based on the result from the Monte Carlo simulation, presence of SB3-14 self-assembly network at the water/hexane interface resulted in the formation of polyamide network with higher crosslinking density, smaller pore sizes, and narrower pore size distribution compared with the conventional IP, but not as good as the SDS self-assembly network.

2.7.4.3. XPS chemical characterization of PA active layer from SARIP (with SB3-14)

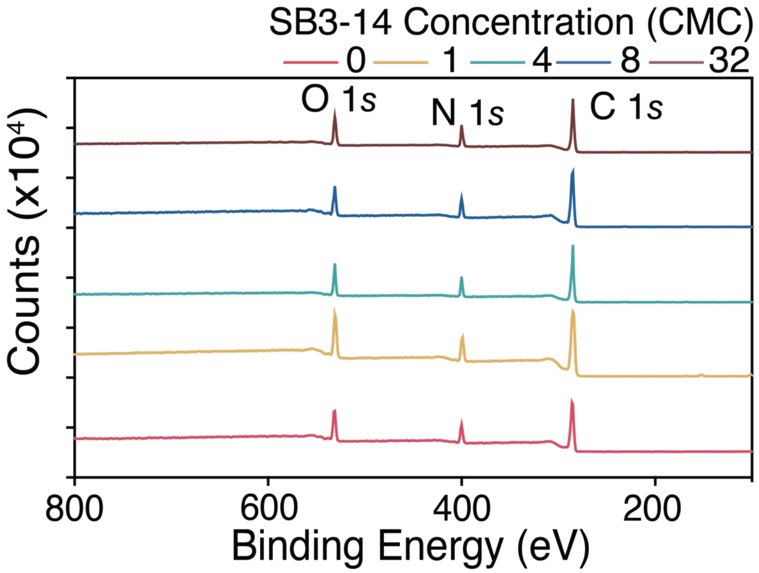

Supplementary Figure 24. XPS survey of polyamide active layer prepared from SARIP as a function of SB3-14 concentration.

2.7.4.3.1. Calculation of degree of cross-linking of PA active layer

Supplementary Table 14. The elemental composition and crosslinking degree of polyamide network from SARIP with SB3-14.

SB3-14 Concentration (CMC)	C(%)	N(%)	O(%)	S(%)	Br(%)	Degree of crosslinking
0	70.24	13.63	15.96	0.17	0	0.76
1	71.56	12.94	15.3	0.19	0	0.75
4	73.24	12.06	14.29	0.41	0	0.75
8	72.63	12.58	14.43	0.36	0	0.79
32	71.58	12.9	15.04	0.48	0	0.77

2.7.4.4. Cross-sectional TEM images of TFC-PA membrane from SARIP (with SB3-14)

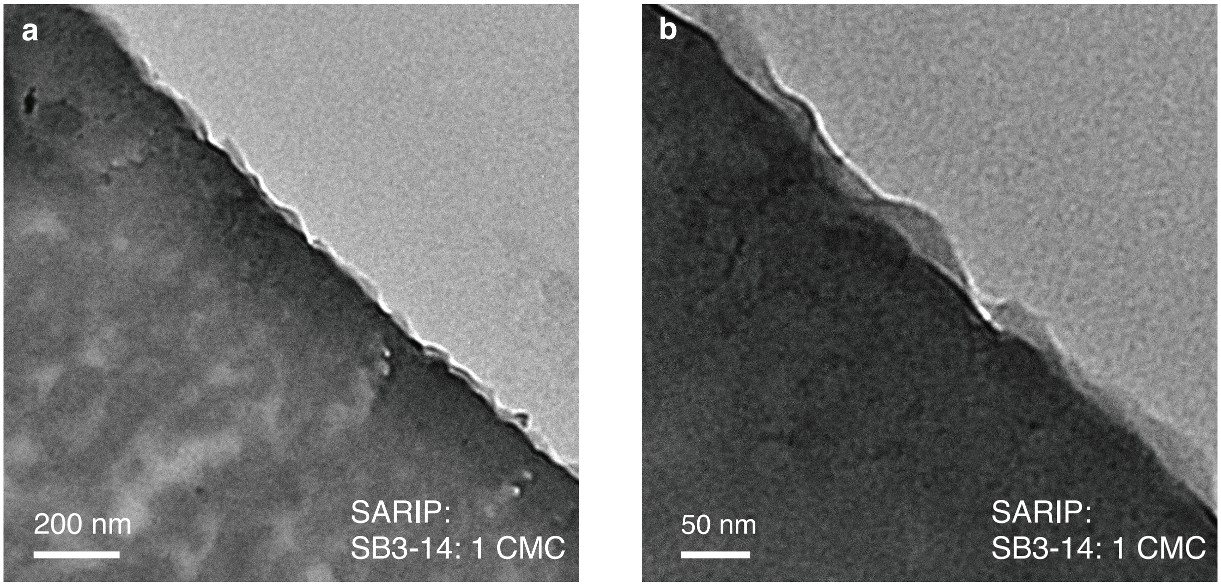

Supplementary Figure 25. Cross-sectional TEM images of TFC-PA membrane prepared from SARIP with SB3-14 (concentration: 1 CMC)

2.7.5. Surface streaming potential of TFC-PA membrane from conventional IP and SARIP

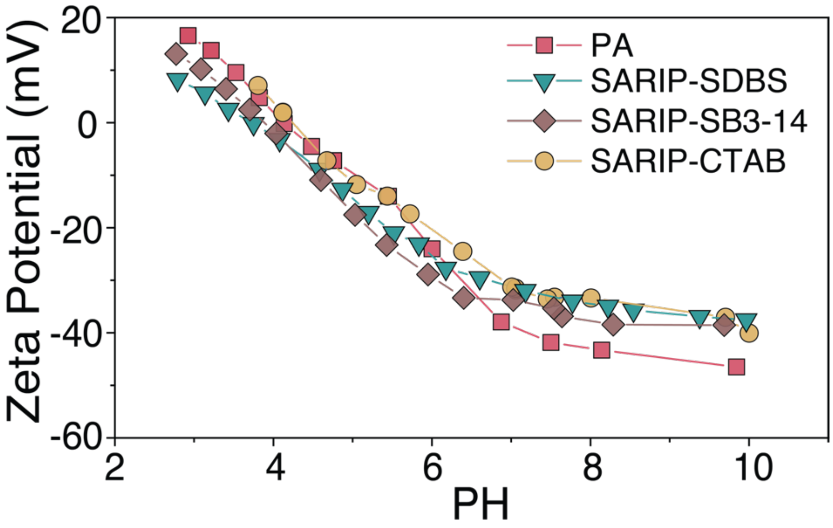

Supplementary Figure 26. Surface streaming potential of TFC-PA membranes from conventional IP and SARIP with different surfactants. (CMC concentration for each surfactant)

2.7.6. Pure water permeability of TFC-PA membrane from conventional IP and SARIP

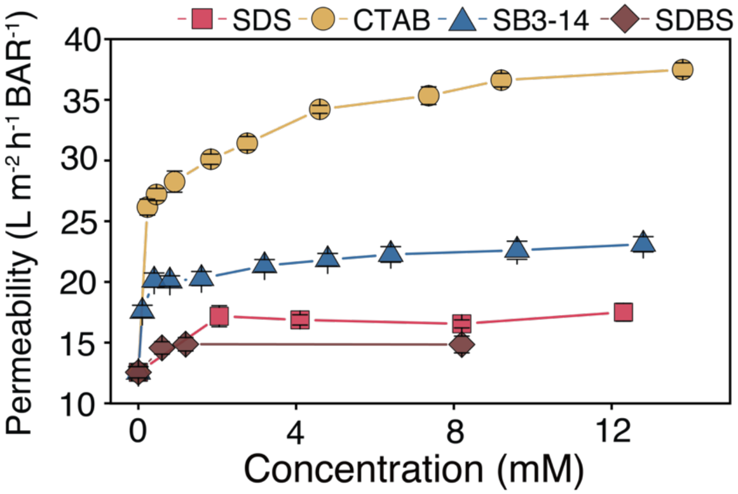

Supplementary Figure 27. Pure water permeability of TFC-PA membranes prepared via conventional IP and SARIP (with surfactants) as a function of surfactant concentrations. Note that the critical micelle concentration for each surfactant is 8.2 mM (SDS), 0.92 mM (CTAB), 0.4 mM (SB3-14) and 1.2 mM (SDBS).

2.7.7. Poly(piperazine-amide) nanofiltration membrane from conventional IP (with sodium p-toluenesulfonate)

2.7.7.1. Interfacial property of Sodium p-tolienesulfonate

Supplementary Table 15. Contact angle (CA) of PIP solution on the PES substrate as a function of sodium p-toluene sulfonate concentration. The addition of sodium p-toluene sulfonate did not have any significant effect on the wettability of PIP solution on the PES substrate.

TS-Na Concentration (mM) (× CMC for SDS)	0	4.1 (0.5)	8.2 (1)	12.3 (1.5)
WCA	43 ± 2	35 ± 2	36± 2	34 ± 2

The addition of sodium p-toluenesulfonate did not change the value of surface tension at different concentrations, indicating that the presence of sulfate groups in the PIP solution did not influence the properties of the interface.

Supplementary Table 16. IFT of hexane and piperazine aqueous solution with the addition of sodium p-toluenesulfonate.

TS-Na Concentration (mM) (× CMC for SDS)	0	4.1 (0.5)	8.2 (1)	12.3 (1.5)
IFT (mN m)	50.92	50.56	50.29	50.17

2.7.7.2. Ion selectivity and permeate flux of TFC-PA membrane from conventional IP (with sodium p-toluenesulfonate)

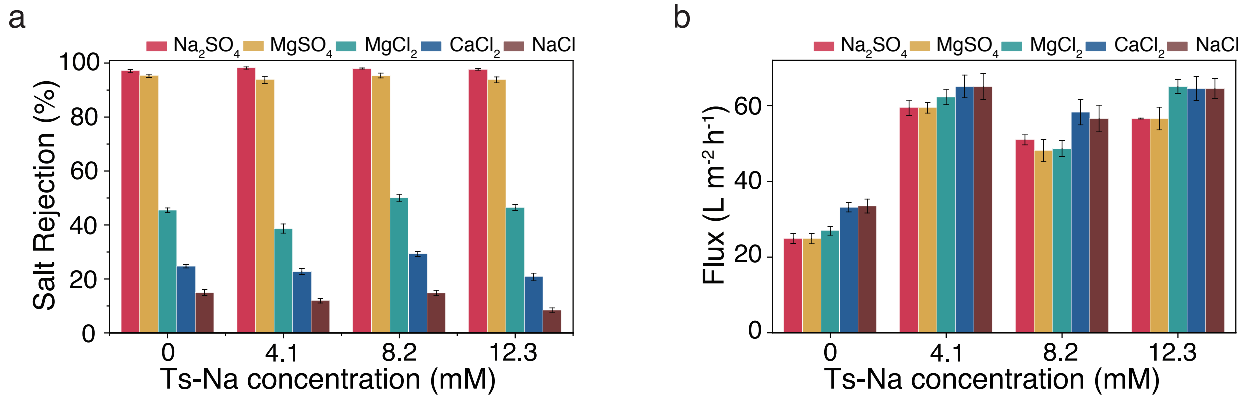

Supplementary Figure 28. (a) Rejection of different solutes by TFC-PA membrane fabricated using conventional IP with different sodium p-toluenesulfonate (Ts-Na) concentrations (b) permeate flux of TFC-PA membrane fabricated using conventional IP with different Ts-Na concentrations. The flux was measured using different feed solution with a hydraulic pressure of 4 bar. The concentrations chosen correspond to 0, 0.5, 1.0, and 1.5 of CMC for SDS.

2.7.7.3. MWCO and pore size of the TFC-PA membrane from conventional IP (with sodium p-toluenesulfonate)

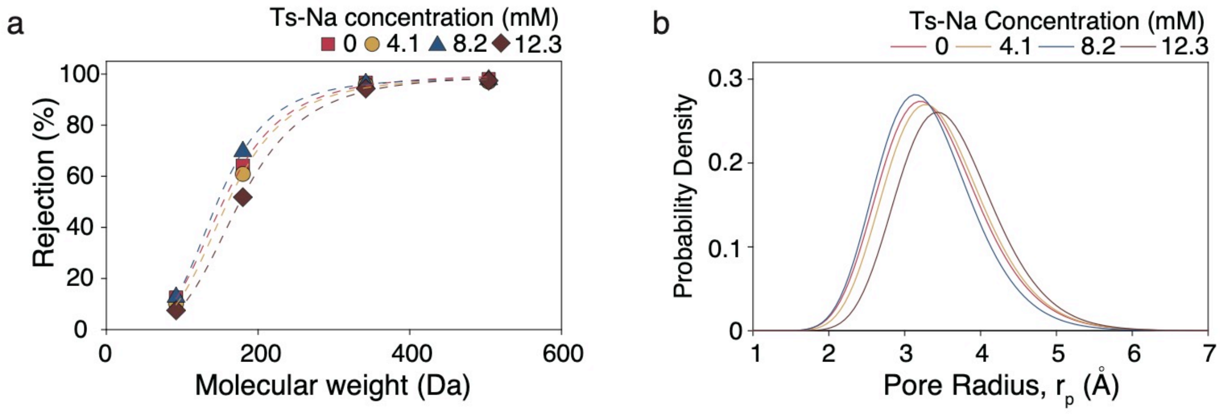

Supplementary Figure 29. (a) Rejection of neutral organic solutes of different MW by TFC-PA membranes fabricated using conventional IP (with Ts-Na) with different Ts-Na concentrations. (b) Pore size distribution estimated with data presented in (a) using Supplementary Equation 4. The concentrations chosen correspond to 0, 0.5, 1.0, and 1.5 of CMC for SDS.

Supplementary Table 17. Mean pore size, standard deviation and MWCO of TFC-PA membrane from conventional IP with different Ts-Na concentrations. (reference concentration: SDS CMC)

Ts-Na Concentration mM (CMC for SDS)	μ_p (nm)	σ_p	MWCO
0	0.334	1.219	274
4.1 (0.5)	0.340	1.212	283
8.2 (1)	0.329	1.211	256
12.3 (1.5)	0.355	1.198	306

In Supplementary Figure 29, the effect of sodium p-toluenesulfonate on the pore size of TFC-PA was negligible, which agrees with the membrane selectivity data in Supplementary Figure 28.

2.7.7.4. XPS chemical characterization of polyamide active layer from conventional IP (with sodium p-toluenesulfonate)

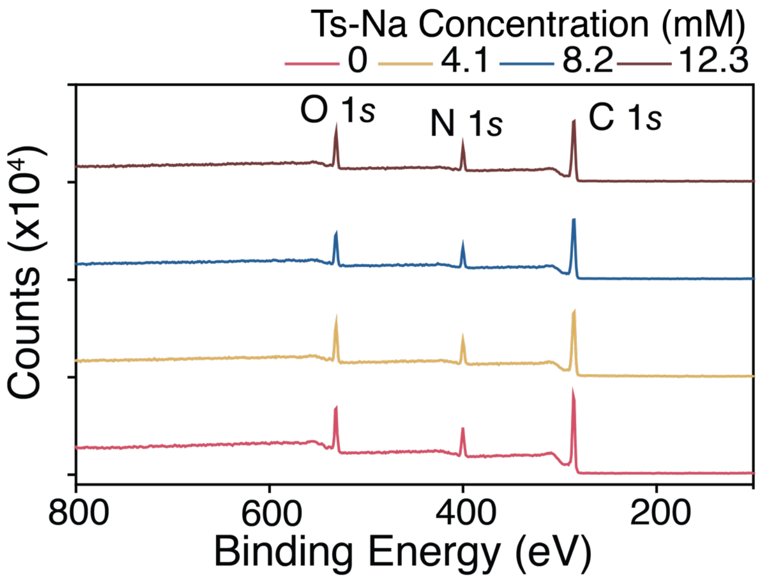

Supplementary Figure 30. XPS survey of polyamide active layer from conventional IP as a function of Ts-Na concentration. (reference concentration: SDS CMC)

Supplementary Table 18. The elemental composition and crosslinking degree of polyamide active layer from conventional IP with Ts-Na. (reference concentration: SDS CMC)

Ts-Na Concentration mM (CMC for SDS)	C(%)	N(%)	O(%)	S(%)	Br(%)	Degree of crosslinking
0	70.24	13.63	15.96	0.17	0	0.76
4.1 (0.5)	71.75	13.49	14.46	0.3	0	0.90
8.2 (1)	73.4	12.76	13.57	0.27	0	0.90
12.3 (1.5)	71.14	13.86	14.76	0.24	0	0.91

2.7.8. Poly(ethylenimine-amide) nanofiltration membrane from conventional IP and SARIP (with SDS)

2.7.8.1. Ion selectivity and permeate flux of the TFC-PA membrane from conventional IP and SARIP (with SDS)

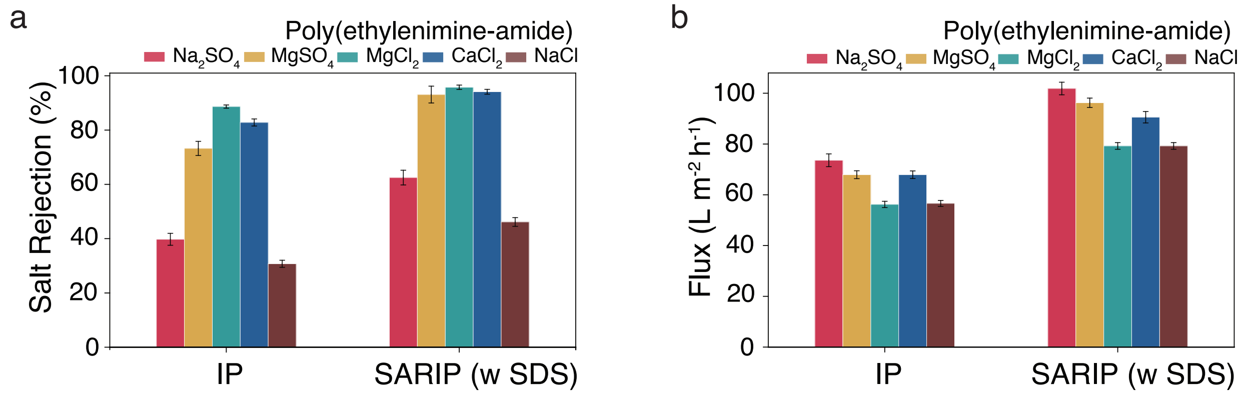

Supplementary Figure 31. Comparison of TFC-PA membranes from conventional IP and SARIP with SDS (1 CMC). (a) solute rejection (b) permeate flux.

The effectiveness of SARIP with SDS on increasing the membrane selectivity was proved in the poly(ethyleneimine-amide) nanofiltration system. An increase of salt rejection of TFC-PA membrane from SARIP with SDS was observed.

2.7.8.2. MWCO and pore size of the TFC-PA membrane from conventional IP and SARIP (with SDS)

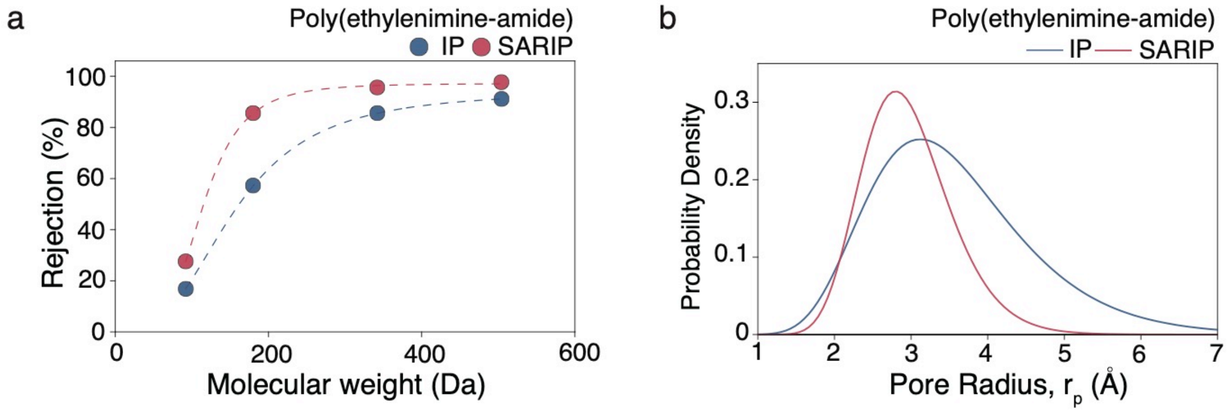

Supplementary Figure 32. Comparison of TFC-PA membranes prepared from conventional IP and SARIP with SDS (1 CMC). (a) MWCO and (b) pore size distribution.

Supplementary Table 19. Mean pore size, standard deviation and MWCO of the TFC-PA from conventional IP and SARIP with SDS (1 CMC) (corresponding to Supplementary Figure 32 a,b).

Poly(ethylenimine-amide)	μ_p (nm)	σ_p	MWCO
IP	0.341	1.345	449
SARIP	0.291	1.217	203

The TFC-PA membrane prepared via SARIP with SDS had much smaller pores than the TFC-PA membrane prepared via conventional IP: the MWCO was reduced to more than one half of the value of the conventional TFC-PA membrane, i.e., the mean pore size decreased from 0.34 nm to 0.29 nm and the distribution of pore size also decreased from 1.345 to 1.217. This provided another evidence of the role of self-assembled SDS dynamic network on the formation of polyamide with smaller and more uniform pore sizes.

2.7.8.3. XPS chemical characterization of poly(ethyleneimine-amide) active layer from conventional IP and SARIP (with SDS)

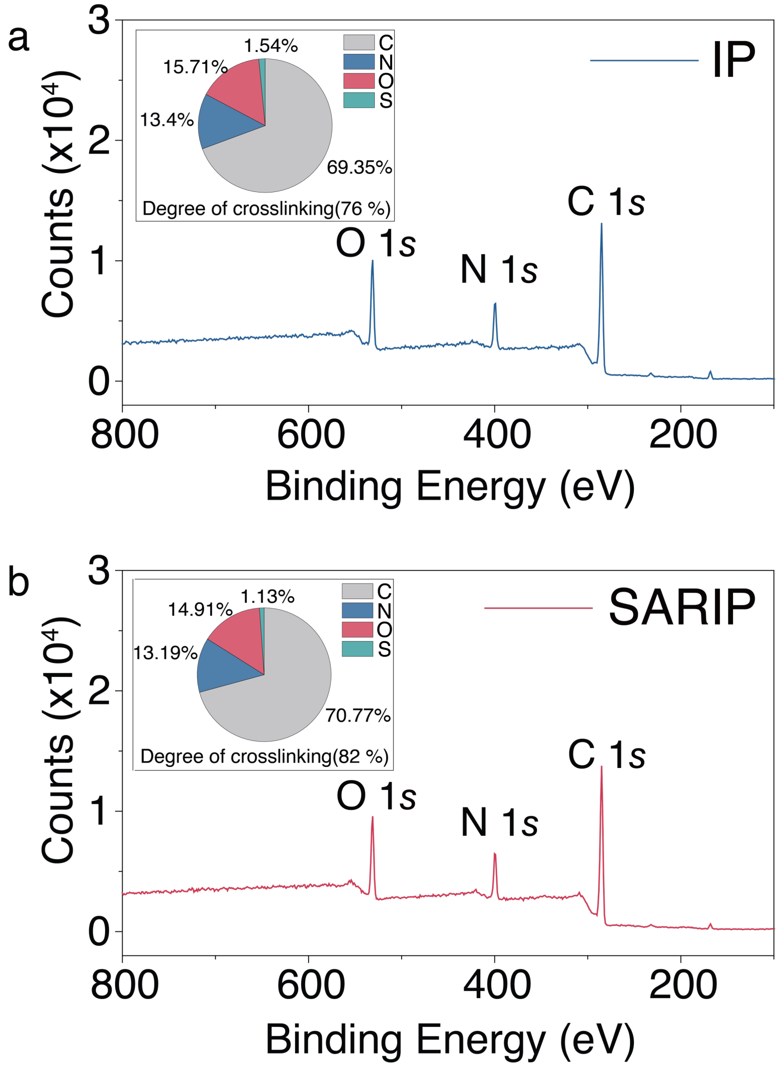

Supplementary Figure 33. XPS survey of poly(ethyleneimine-amide) active layers (a) from conventional IP. (b) from SARIP with SDS (1 CMC). The presence of the SDS self-assembled network promoted the formation of the PA network with a higher crosslinking degree.

2.8. TFC-PA NF membrane prepared via SARIP with 2 CMC SDS

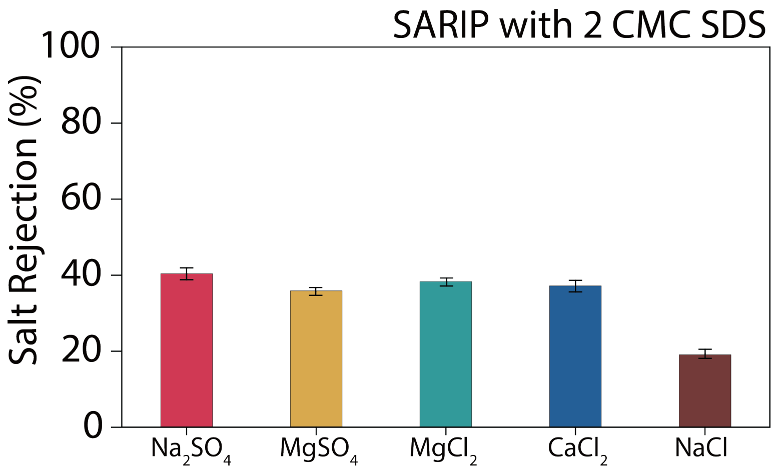

Supplementary Figure 34. Rejection of different solutes by TFC-PA membranes fabricated using SARIP with 2 CMC SDS.

2.9. MWCO and pore size of TFC-PA membrane from SARIP (with SDS)

Supplementary Table 20. Mean pore size, standard deviation and MWCO of TFC-PA membrane from SARIP with different SDS concentrations (corresponding to Figure 4b and inset)

SDS concentration (CMC)	μ_p *(nm)*	σ_p	*MWCO*
0	0.334	1.219	274
0.25	0.310	1.177	208
0.5	0.295	1.156	178
1	0.290	1.144	167
1.5	0.300	1.180	195

Increasing PIP concentration in conventional IP results in polyamide with smaller mean pore size and MWCO. However, the standard deviation remains nearly unchanged (Supplementary Table 3), i.e., the pore size is still widely distributed. In contrast, TFC-PA from SARIP does not only have a smaller mean pore size and MWCO but also a smaller standard deviation (Supplementary Table 7).

3. Supplementary Reference:

1. Hung, W. S., Lo, C. H., Cheng, M. L., Chen, H., Liu, Chakka, L., Nanda, D., Tung, K. L., Huang, S. H., Lee, K. R., Lai, J. Y., Sun, Y. M., Yu, C. C., Zhang, R. & Jean, Y. C. Polymeric membrane studied using slow positron beam. *Appl. Surf. Sci.* **255**, 201–204 (2008).
2. Lau, W. J., Ismail, A. F., Misdan, N. & Kassim, M. A. A recent progress in thin film composite membrane: a review. *Desalination* **287**, 190–199 (2012).
3. Petersen, R. J. Composite reverse osmosis and nanofiltration membranes. *J. Membr. Sci.* **83**, 81–150 (1993).
4. Chen, G. E., Liu, Y. J., Xu, Z. L., Tang, Y. J., Huang, H. H. & Sun, L. Fabrication and characterization of a novel nanofiltration membrane by the interfacial polymerization of 1,4-diaminocyclohexane (DCH) and trimesoyl chloride (TMC). *RSC Adv.* **5**, 40742–40752 (2015).
5. Nightingale. Jr., E. R. Phenomenological theory of ion solvation. Effective radii of hydrated ions. *J. Phys. Chem.* **63**, 1381–1387 (1959).
6. Marcus, Y. Thermodynamics of solvation of ions. *J. Chem. Soc., Faraday Trans.* **89**, 713–718 (1993).
7. Donnan, F. G. Theory of membrane equilibria and membrane potentials in the presence of non-dialysing electrolytes. A contribution to physical-chemical physiology. *J. Membr. Sci.* **100**, 45–55 (1995).
8. Peeters, J. M. M., Boom, J. P., Mulder, M. H. V. & Strathmann, H. Retention measurements of nanofiltration membranes with electrolyte solutions. *J. Membr. Sci.* **145**, 199–209 (1998).
9. Epsztein, R., Shaulsky, E., Dizge, N., Warsinger, D. M. & Elimelech, M. Role of ionic charge density in donnan exclusion of monovalent anions by nanofiltration. *Environ. Sci. Technol.* **52**, 4108–4116 (2018).
10. Tang, C. Y., Kwon, Y. N. & Leckie, J. O. Effect of membrane chemistry and coating layer on physiochemical properties of thin film composite polyamide RO and NF membranes. I. FTIR and XPS characterization of polyamide and coating layer chemistry. *Desalination* **242**, 149–167 (2009).

11. Beverly, S., Seal, S. & Hong, S. Identification of surface chemical functional groups correlated to failure of reverse osmosis polymeric membranes. *J. Vac. Sci. Technol. A* **18**, 1107–1113 (2000).
12. Ariza, M. J., Benavente, J., Rodriguez-Castellon, E. & Palacio, L. Effect of hydration of polyamide membranes on the surface electrokinetic parameters: surface characterization by X-ray photoelectronic spectroscopy and atomic force microscopy. *J. Colloid Interface Sci.* **247**, 149–158 (2002).
13. Akin, O. & Temelli, F. Probing the hydrophobicity of commercial reverse osmosis membranes produced by interfacial polymerization using contact angle, XPS, FTIR, FE-SEM and AFM. *Desalination* **278**, 387–396 (2011).
14. France, R. M. & Short, R. D. Plasma treatment of polymers: the effects of energy transfer from an argon plasma on the surface chemistry of polystyrene, and polypropylene. A high-energy resolution X-ray photoelectron spectroscopy study. *Langmuir* **14**, 4827–4835 (1998).
15. Eastoe, J. & Dalton, J. S. Dynamic surface tension and adsorption mechanisms of surfactants at the air–water interface. *Adv. Colloid Interface Sci.* **85**, 103–144 (2000).
16. Matijević, E. & Pethica, B. A. The properties of ionized monolayers. Part 1.—Sodium dodecyl sulphate at the air/water interface. *Trans. Faraday Soc.* **54**, 1382–1389 (1958).
17. Elworthy, P. H. & Mysels, K. J. The surface tension of sodium dodecylsulfate solutions and the phase separation model of micelle formation. *J. Colloid Interface Sci.* **21**, 331–347 (1966).
